# Tracking the origin of two genetic components associated with transposable element bursts in domesticated rice

**DOI:** 10.1038/s41467-019-08451-3

**Published:** 2019-02-07

**Authors:** Jinfeng Chen, Lu Lu, Jazmine Benjamin, Stephanie Diaz, C. Nathan Hancock, Jason E. Stajich, Susan R. Wessler

**Affiliations:** 1 Department of Microbiology and Plant Pathology, University of California, Riverside, CA 92521 USA; 2 Department of Botany and Plant Sciences, University of California, Riverside, CA 92521 USA; 3 Institute for Integrative Genome Biology, University of California, Riverside, CA 92521 USA; 4 Department of Biology and Geology, University of South Carolina Aiken, Aiken, SC 29801 USA

## Abstract

Transposable elements (TEs) shape genome evolution through periodic bursts of amplification. In this study prior knowledge of the *mPing/Ping/Pong* TE family is exploited to track their copy numbers and distribution in genome sequences from 3,000 accessions of domesticated *Oryza sativa* (rice) and the wild progenitor *Oryza rufipogon*. We find that *mPing* bursts are restricted to recent domestication and is likely due to the accumulation of two TE components, *Ping16A* and *Ping16A_Stow*, that appear to be critical for *mPing* hyperactivity. *Ping16A* is a variant of the autonomous element with reduced activity as shown in a yeast transposition assay. Transposition of *Ping16A* into a *Stowaway* element generated *Ping16A_Stow*, the only *Ping* locus shared by all bursting accessions, and shown here to correlate with high *mPing* copies. Finally, we show that sustained activity of the *mPing/Ping* family in domesticated rice produced the components necessary for *mPing* bursts, not the loss of epigenetic regulation.

## Abstract (web summary)

Transposable element (TE) bursts shape genome evolution but their origin remains unclear. Here, the authors show that a burst is restricted to only a few domesticated rice accessions and is associated with the acquisition of two TE variants, Ping16A and Ping16A_Stow, not the loss of TE silencing.

## Introduction

Eukaryotic genomes are populated with transposable elements (TEs), many attaining copy numbers of hundreds to thousands of elements by rapid amplification, called a TE burst^1^. For a TE to successfully burst, it must be able to increase its copy number without killing its host or being silenced by host surveillance^2,3^. However, because the vast majority of TE bursts have been inferred after the fact—via computational analysis of whole-genome sequence—the stealth features they require for success have remained largely undiscovered^2,4^.

**Fig. 1:**
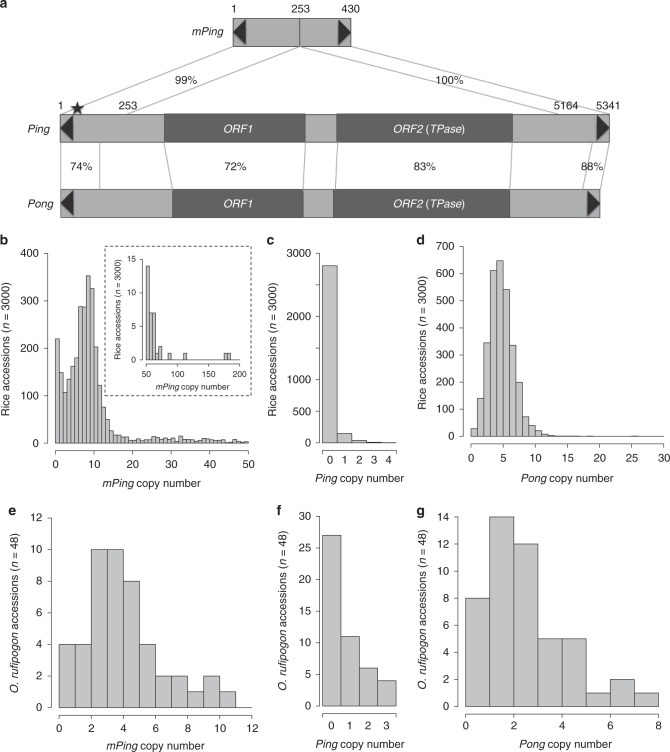
Abundance of *mPing*, *Ping*, and *Pong* elements in rice and *O. rufipogon*. **a** Comparison of structures of *mPing*, *Ping,* and *Pong*. TIRs are indicated by black triangles. Two protein-coding genes *ORF1* and *ORF2* (*TPase*) encoded by *Ping* or *Pong* are indicated by dark gray boxes. Homologous regions between elements are connected by lines and percent identities are shown. The black star on *Ping* indicates the + 16 G/A SNP that differs between *mPing* and *Ping16A*. Copy numbers across the 3000 rice accessions of *mPing* (**b**), *Ping* (**c**), and *Pong* (**d**). The bar plot in the dashed box in **b** shows accessions with >50 *mPing* elements. **e**, *mPing* copy number of 48 *O. rufipogon* accessions. **f** *Ping* copy number of 48 *O. rufipogon* accessions. **g** *Pong* copy number of 48 *O. rufipogon* accessions. Source data for Fig. 1b–g are provided in Supplementary Data 1-2

Revealing these features requires the identification of a TE in the midst of a burst. This was accomplished for the miniature inverted-repeat TE (MITE) *mPing* from rice^5,6^. MITEs are nonautonomous DNA transposons that are the most common TE associated with the noncoding regions of plant genes^1^. To understand how MITEs attain high copy numbers, a computational approach was used to identify *mPing*, and its source of transposase, encoded by the related autonomous *Ping* element (Fig. 1a)^5^.

Ongoing bursts of *mPing* were discovered in four temperate *japonica* accessions: EG4, HEG4, A119, and A123, whose genomes were sequenced, and insertion sites and epigenetic landscape determined^6–8^. These analyses uncovered two features of successful *mPing* bursts. First, although *mPing*, like other DNA TEs, prefers genic insertions, de novo insertions in exons were only 14% of expected for random insertions, thus minimizing harm to the host^6,8^. Exon avoidance arises from *mPing*’s extended 9-bp adenine-thymine (AT)-rich insertion preference^6,8^ coupled with rice exon sequences that are significantly more guanine-cytosine (GC) rich than rice introns (51% vs. 37%)^9^. Second, because *mPing* does not share coding sequences with *Ping* (Fig. 1a), increases in its copy number and host recognition of its sequences does not silence *Ping* genes, thus allowing the continuous production of the proteins necessary to sustain the burst for decades^7^.

The contributions of two other genetic components to the success of the bursts could not be assessed previously and are a focus of this study. First, all *Ping* elements in the four bursting accessions contain a single nucleotide polymorphism (SNP) at position 16 ( + 16 G/A) that distinguishes *mPing* and *Ping* sequences (Fig. 1a). The second genetic component is a single *Ping* locus (called *Ping16A_Stow*) that is the only *Ping* locus shared by all bursting accessions^7^. Comparative sequence analysis of two of the four bursting accessions (A123 and A119) revealed that they were derived by self or sibling pollination about a century ago from a common ancestor that had not yet undergone *Ping* or *mPing* amplification^7^. Significantly, this common ancestor had only a single *Ping* locus, which was *Ping16A_Stow*^7^.

To understand the origin of these genetic components and their possible role in the burst, we analyzed the presence, sequence, and copy numbers of *Ping* and *mPing* elements in the genomes of 3000 domesticated rice accessions and 48 genomes of their wild progenitor, *Oryza rufipogon*. Rice has been divided into five major subgroups (*indica*, *aus*/*boro*, *aromatic*, temperate *japonica*, and tropical *japonica*) that are thought to have originated from distinct populations of the wild progenitor *O. rufipogon* that arose prior to domestication^10,11^. Rice genomes are very stable: all analyzed genomes are composed of 12 chromosomes^12,13^, and rice subgroups share high sequence identity ( > 98.9%)^13^. However, the genomes also exhibit extensive presence–absence variation both within (5%) and between (10%–19%) subgroups^13,14^, with TEs representing more than half of this variation. In addition, significant gene flow from *japonica* to *indica* and *aus* has been noted previously, reflecting the more ancient origin of *japonica*^10,15^.

Knowledge of the relationships between the major subgroups of rice and the populations of *O. rufipogon* have been utilized in this study to better understand the identity and origin of the components necessary for *mPing* bursts. Of particular interest was whether (i) *mPing* bursts could be detected in other accessions of wild and/or domesticated rice, (ii) the + 16 G/A *Ping* SNP and *Ping16A_Stow* could be detected in wild rice or first appeared in domesticated rice, and (iii) the presence of + 16 G/A *Ping* SNP and *Ping16A_Stow* correlated with higher *mPing* copy numbers.

Finally, another potential player that may be implicated in *mPing* bursts, *Pong*, a related transposase-encoding element, is a focus of this study (Fig. 1a). The *Pong* element is the closest relative of *Ping* and there are at least five identical copies found in the genome of all rice accessions analyzed to date^7,16^. *Pong* encoded proteins catalyzed the transposition of *mPing* in rice cell culture^5^ and in transposition assays in *Arabidopsis thaliana* and yeast^17,18^. However, *Pong* elements do not catalyze *mPing* transposition in planta because all *Pong* copies are effectively silenced and its sequences are associated with heterochromatin^7^. Here we are able to address questions regarding the origin and stability of *Pong* silencing before and after domestication.

Our analysis show that *mPing* copy number has burst only in a few domesticated accessions and is associated with the acquisition of two variants of the transposase loci, *Ping16A* and *Ping16A_Stow*. The proportion of accessions with *Ping16A* has increased in domesticated rice while the original *Ping* (*Ping16G*) has been dramatically reduced. A transposition event of *Ping16A* into a *Stowaway* element created *Ping16A_Stow* whose presence correlates with accessions that have high *mPing* copies. We reject the hypothesis that a loss of global epigenetic regulation has occurred as no other TEs have amplified, indicating that these new *Ping* loci are the primary driver of the observed *mPing* burst in domesticated rice.

## Results

### Detection of *mPing, Ping*, and *Pong* element

Insertion sites and copy numbers for *mPing*, *Ping*, and *Pong* were identified from genome sequences of 3000 rice accessions using RelocaTE2^19^ (see Methods). The paired-end DNA libraries had an average insert size of ~ 500 bp and were sequenced to a depth of 14-fold genome coverage^20^, which allowed clear distinction between *mPing, Ping, and Pong* elements (Fig. 1a). Sequence analyses identified a total of 27,535 *mPings*, 262 *Pings*, and 12,748 *Pongs* (Figs. 1b–d and Supplementary Data 1). Copy numbers of *mPing*, *Ping*, and *Pong* elements in each genome were also estimated using a read depth method (see Methods). Outputs from the RelocaTE2 and read depth methods were well correlated (Pearson’s correlation, *R* = 0.97, *P* < 2.2e–16 for *mPing*; *R* = 0.82, *P* < 2.2e–16 for *Ping*; *R* = 0.66, *P* < 2.2e–16 for *Pong*; Supplementary Figure 1) indicating that both methods to estimate approximate *mPing*, *Ping*, and *Pong* copy numbers in the 3000 rice accessions were robust. Insertion sites and copy numbers for *mPing*, *Ping*, and *Pong* were also identified for 48 *O. rufipogon* accessions, but only the read depth method was used because of the limited insert size of the libraries (Supplementary Data 2). In total, 195 *mPings*, 25 *Pings*, and 125 *Pongs* were estimated to be present in the 48 *O. rufipogon* accessions (Supplementary Data 2, Figs. 1e–g, and Supplementary Figure 2).

### Copy number variation of *mPing* and *Ping* elements

None of the 3000 rice accessions analyzed in this study have more *mPing* elements than the 231–503 copies found in the four temperate *japonica* accessions (HEG4, EG4, A119, A123) in the midst of *mPing* bursts^7^. Of the 3000 rice accessions, 2780 (92.7%) contain *mPing*, with an average of about 9 elements per accession (Fig. 1b). Temperate *japonica* accessions do, however, have significantly more *mPing* elements (~30.5/accession) than tropical *japonica* (~2.6/accession), *indica* (~8.2/accession), or *aus/boro* (~3.8/accession) (one-way analysis of variance (ANOVA) with Tukey’s honest significant difference (HSD) test, adjusted *P*-value < 2e–16; Supplementary Table 1 and Supplementary Figure 3). All *O. rufipogon* accessions have *mPing* elements with copy numbers ranging from 1 to 11 (mean = 4.06, standard deviation = 2.39; Fig. 1e and Supplementary Figure 2).

Prior studies identified four subtypes of *mPing* elements (*mPingA-D*) in domesticated rice (Supplementary Figure 4)^5^, representing four distinct deletion derivatives of *Ping*. Two of the four subtypes (*mPingA*,*B*) were previously detected in *O. rufipogon* accessions^21,22^. Here we detected all four subtypes of *mPing* elements in *O. rufipogon* accessions (Supplementary Table 2) indicating that *mPingA-D* arose in *O. rufipogon* prior to domestication.

**Fig. 2:**
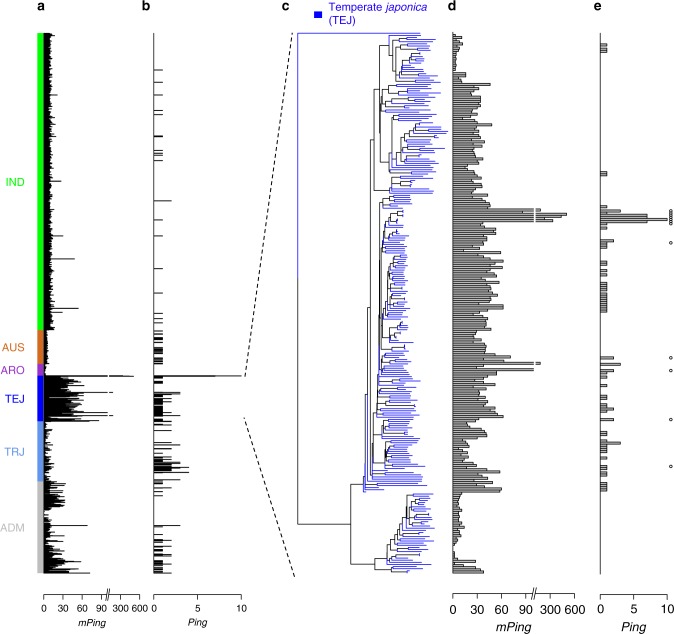
Copy numbers of *mPing, Ping*, and *Pong* elements in rice subgroups. **a** *mPing* copy numbers in 3000 genomes and the four accessions undergoing *mPing* bursts (HEG4, EG4, A119, and A123). Colors represent the five major rice subgroups: *indica* (IND), *aus/boro* (AUS), *aromatic* (ARO), temperate *japonica* (TEJ), tropical *japonica* (TRJ), and admixed (ADM). **b** *Ping* copy numbers in 3000 genomes and the four accessions undergoing *mPing* bursts. **c** Neighbor-joining tree of temperate *japonica* accessions using genome-wide SNPs. **d** *mPing* copy number of temperate *japonica* accessions. **e** *Ping* copy number of temperate *japonica* accessions. Accessions that have the *Ping16A_Stow* locus are noted with open circles. Source data for Fig. 2a, b, d, e are provided in Supplementary Data 1

Like *mPing*, none of the 3000 genomes analyzed in this study have more *Ping* elements (7–10) than the four accessions undergoing *mPing* bursts^7^. *Ping* elements were detected in only 199 of 3000 accessions (6.6%) (Fig. 2 and Table 1) with most of the 199 (74.8%) having only a single copy and two accessions having 4 *Pings* (Fig. 2b). In contrast, *Ping* elements were detected in 21 of 48 (43.7%) of the *O. rufipogon* accessions analyzed (Table 1 and Supplementary Figure 2). These data suggest that it is likely that *Ping* was selected against or lost from most accessions during the hypothesized two or more domestication events from *O. rufipogon* populations^10,14^.

**Table 1:** Distribution of *Ping* variants and *Ping16A_Stow* genotypes in domesticated rice and *O. rufipogon*

Subgroups	Number of accessions	Number of accessions with *Ping*^a^	*Ping* variants: *Ping16G*	*Ping* variants: *Ping16A*	*Ping16A_Stow*: *Stowaway* only	*Ping16A_Stow: Stowaway* with *Ping*
*O. sativa*	3000	199 (6.6%)	31	154	188	11
* -indica*	1651	20 (1.2%)	8	9^b^	10	0
* -aus/boro*	189	28 (14.8%)	19	0	0	0
* -*temperate *japonica*	250	61 (24.4%)	1	61	121	8
* -*tropical *japonica*	335	51 (15.2%)	0	51	2	0
* -aromatic*	65	0 (0%)	0	0	0	0
* -*admixed	510	39 (7.6%)	3	33^c^	55	3
*O. rufipogon*	48	21 (43.7%)	21	0	4	0
* -Or-I*	13	7 (53.8%)	7	0	0	0
* -Or-II*	23	10 (43.4%)	10	0	1	0
* -Or-IIIa*	6	2 (33.3%)	2	0	3	0
* -Or-IIIb*	6	2 (33.3%)	2	0	0	0

*Ping16A_Stow* is defined as a locus where *Ping* has inserted into the *Stowaway* element on chromosome 1 (2640500–2640502)

^a^“Number of accessions with *Ping16G*” plus “Number of accessions with *Ping16A*” is less than or equal to “Number of accessions with *Ping*” because *Ping* genotypes in some accessions cannot be determined from available sequences. An exception is “temperate *japonica*”, where one accession (IRIS_313-10564) has both *Ping16G* (Chr8: 2964281–2964283) and *Ping16A* (Chr6: 23521641–23526981)

^b^Eight *indica* accessions have *Ping16A* that are located in regions showing evidence of introgression from *japonica* (seven accessions share the locus Chr3: 21965880–21965882 and one accession has the Nipponbare *Ping* locus Chr6: 23521641–23526981). One *indica* accession has *Ping16A* in a region with *indica* background. Analyses were performed with RFMix v2.03

^c^Thirty-one admixed accessions have *Ping16A* from *japonica*. Two admixed accessions have *Ping16A* that are located in regions with ambiguous origin. Analyses were performed with RFMix v2.03

### Origin of a *Ping* variant and its possible significance

**Fig. 3:**
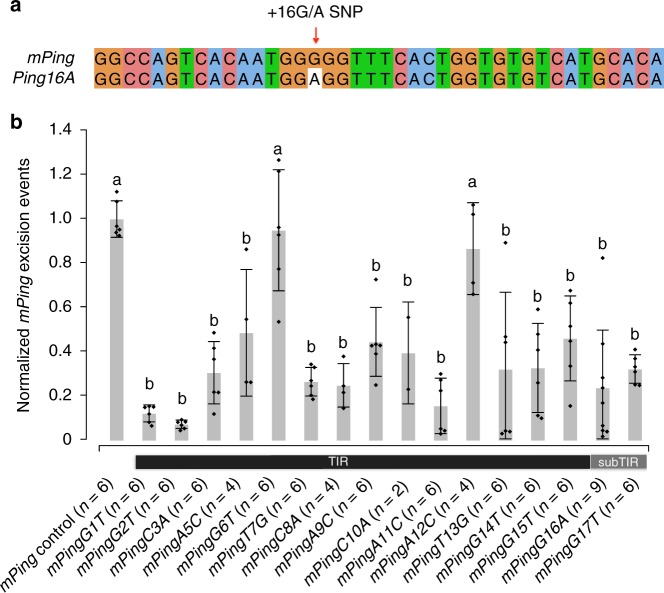
Transposition frequency of *mPing* variants in the yeast assay. **a** Sequence alignment of *mPing* and *Ping16A* terminal sequence (1–40 bp). The SNP between *mPing* and *Ping16A* at position 16 ( + 16 G/A SNP) is indicated by the red arrow. **b** Transposition frequency of *mPing* variants with mutations at the 5ʹ end in the yeast assay. *X* axis indicates *mPing* variants with mutations at 14 positions in the 5ʹ TIR and two positions outside the TIR. For example, *mPingG16A* represents an *mPing* variant having a G-to-A mutation at position 16. A variant *mPingC4A* was not included because the lack of qualified experiments. *Y* axis shows transposition frequency that was measured as *mPing* excision events per million cells and normalized to the control *mPing*. The error bars show standard deviation (s.d.) of 2-9 independent biological replicates. Letters (a and b) above the bars indicate significant differences of transposition frequency between *mPing* variants and control (adjusted *P*-value ≤ 0.05). The adjusted *P-*values are based on a one-way ANOVA (*P-*value = 2.37e–15, *F-*value = 12.34, DF = 16) followed by a Tukey’s honest significant difference (Tukey’s HSD) test. Source data for Fig. 3b are provided as a Source Data file

Analysis of the extensive collection of rice genomes revealed that a SNP distinguishing *Ping* and *mPing* ( + 16 G/A), located adjacent to the 15-bp terminal inverted repeat (TIR) (Fig. 3a), may be implicated in *mPing* bursts. *Pings* having these SNPs are distinguished herein as *Ping16G* (identical shared sequences with *mPing*) and *Ping16A*. First, all 21 *O. rufipogon* accessions with *Ping* have only *Ping16G*, which has the same sequence at + 16G/A as *mPing* (Table 1). Thus, *Ping16G* is the original *Ping* and all four *mPing* subtypes (*mPingA-D*, Supplementary Table 2) arose prior to domestication by internal deletion. Second, of the 199 domesticated rice accessions with *Ping*, 31 have *Ping16G*, whereas 154 have *Ping16A* (Table 1). The presence of the derived *Ping16A* in both *indica* and *japonica* accessions was initially confusing as it suggested the unlikely scenario that this variant arose independently during or after the hypothesized two domestication events that led to these subspecies^10,14^. However, closer examination of local sequence ancestry revealed that, where a determination could be made, all of the *Ping16A* loci in *indica* and admixed accessions originated by introgression from *japonica* (Table 1). Thus, *Ping16A* has experienced limited but significant proliferation during or after *japonica* domestication such that it now accounts for the majority of *Ping* elements present in domesticated rice accessions (Table 1).

### Reduced mobility of *Ping16A* in yeast assays

The TIRs and adjacent sequences of several DNA transposons have been shown to be functionally significant with mutations of these sequences reducing transposition frequency by decreasing the binding of transposase^23,24^. Because the SNP distinguishing *Ping16A* from *Ping16G* is adjacent to the 15-bp 5ʹ TIR (Fig. 3a), we employed a yeast assay to assess transposition rates of 14 mutations within and 2 mutations adjacent to the 5ʹ TIR (Fig. 3b). In this assay, *Pong* transposase and an enhanced *Ping ORF1* (the putative binding domain) catalyzes transposition of *mPing* inserted in an *ADE2* reporter gene, thereby allowing growth of yeast cells^18,25^. The results indicate that both the mutations adjacent to the TIRs (G16A and G17T) and 12 of 14 mutations in the TIR significantly reduced *mPing* transposition (one-way ANOVA with Tukey’s HSD test, adjusted *P*-value ≤ 0.05; Fig. 3b), supporting the hypothesis that this SNP ( + 16 G/A) may have functional significance by reducing *Ping16A*’s mobility. Although *Pong* transposase, which was shown previously to catalyze higher transposition frequency than *Ping*, was used in this experiment to facilitate the yeast transposition assays, its catalytic mechanism is likely indistinguishable from *Ping* transposase^25^. Furthermore, the reduced transposition of the G16A mutant (*mPingG16A*) was independently confirmed using *Ping* transposase (Supplementary Figure 5).

### A *Ping* locus correlates with higher *mPing* copy number

**Fig. 4:**
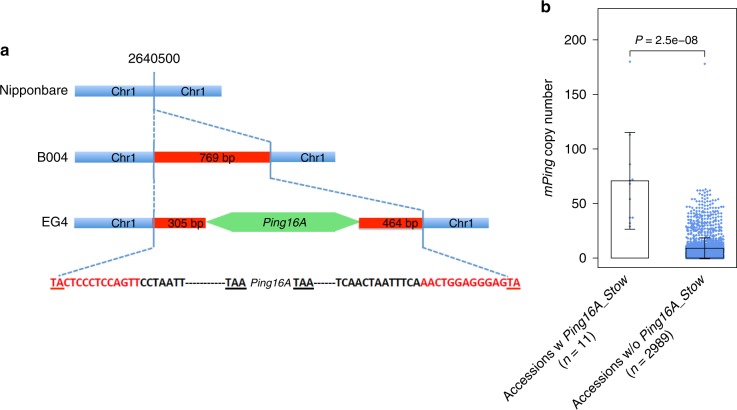
Structure of the *Ping16A_Stow* locus, which is associated with increased *mPing* copy number. **a** Structure of the *Ping16A_Stow* insertion site. The *Ping16A* element (green arrow) is inserted in the middle of a nonautonomous *Stowaway* element (red box), which is not in Nipponbare (blue bar). Nucleotides shown within the blue dotted lines are the sequences of the nonautonomous *Stowaway* element. Target site duplications (TSDs) are indicated by nucleotides underscored. **b** Comparisons of *mPing* copy number in 3000 rice accessions with or without *Ping16A_Stow* in the genome. Gray dots indicate *mPing* copy number of rice accessions in each category. The error bars show standard deviation (s.d.) of each category. Differences in *mPing* copy numbers between two categories were tested by a two-tailed Wilcoxon–Mann–Whitney test. Source data for Fig. 4b are provided in Supplementary Data 1

The four accessions previously shown to be undergoing *mPing* bursts (HEG4, EG4, A119, A123) have many (7–10) *Pings*, and all share only a single *Ping*, *Ping16A_Stow*^7^. This correlation suggests that acquisition of *Ping16A_Stow* may have initiated the burst. *Ping16A_Stow*, located on chromosome 1 (2640500–2640502), is comprised of the *Ping16A* variant inserted in a 769-bp *Stowaway* element (Fig. 4a). Of interest was whether any of the 3000 accessions had *Ping16A_Stow* and, if so, did they also have more *mPings*.

Among the 3000 accessions, 11 have *Ping16A_Stow* (188 have only the *Stowaway* insertion at this locus) (Table 1) and these accessions have significantly more *mPings* (two-tailed Wilcoxon–Mann–Whitney test, *P* = 2.5e–08; Fig. 4b, Table 2, and Supplementary Table 3), providing additional correlative evidence for the involvement of *Ping16A_Stow* in *mPing* bursts.

**Table 2:** *Ping* copy numbers and genotypes in rice accessions with high copy numbers of *mPing*

Accession^a^	Origin	Subgroups	*mPing* copy number	*Ping* copy number	*Ping* *+* 16G/A SNP genotypes	*Ping16A_Stow*
HEG4^a^	Japan	Temperate *japonica*	503	7	*Ping16A*	Yes
EG4^a^	Japan	Temperate *japonica*	437	7	*Ping16A*	Yes
A123^a^	Japan	Temperate *japonica*	231	10	*Ping16A*	Yes
A119^a^	Japan	Temperate *japonica*	333	7	*Ping16A*	Yes
B160	China	Temperate *japonica*	180	3	*Ping16A*	Yes
IRIS_313-15904	South Korea	Temperate *japonica*	178	3	*Ping16A*	No
B235	China	Temperate *japonica*	113	2	*Ping16A*	Yes
B005	Japan	Temperate *japonica*	86	1	*Ping16A*	Yes
B003	China	Admixed	72	2	*Ping16A*	Yes
B001	China	Temperate *japonica*	71	2	*Ping16A*	Yes

^a^From Lu et al.^7^

### *Pong* has been stably silenced since domestication

*Pong* encoded proteins catalyze transposition of *mPing* in yeast and *A. thaliana* assays^17,18^ and in rice cell culture^5^. However, because *Pong* elements are epigenetically silenced in Nipponbare and in accessions undergoing *mPing* bursts (HEG4, EG4, A119, A123)^7^, there is no evidence to date that *Pong* has an impact on *Ping* or *mPing* copy number or distribution.

Data from this study extend previous findings^7^ and suggest that *Pong* was silenced in *O. rufipogon* and has been stably silenced in domesticated rice. *Pong* elements are present in the genomes of almost all of the analyzed rice accessions (99.1%, 2972/3000), and *Pong* copy numbers vary little within or between subgroups (Supplementary Figure 6). On average, rice accessions have four *Pong* elements (Fig. 1d). All *O. rufipogon* accessions have *Pong* elements (Supplementary Figure 2), except four (W1849, W1850, W2022, W2024), which appear to contain only *Pong* deletion derivatives (see Methods). As in domesticated rice, there is minimal *Pong* copy number variation among the *O. rufipogon* accessions examined (Supplementary Figure 2).

Six rice accessions with higher *Pong* copy numbers (14–25) were analyzed to determine if this resulted from *Pong* activation. First, because active *Pong* elements produce proteins that catalyze *mPing* transposition, we tested if the genomes of these lines contained more *mPings*. However, all six accessions had the same range of *mPing* copies as accessions with few *Pongs* (Supplementary Table 4). Second, because host regulatory mechanisms suppress transposition, other potentially active TEs (elements shown previously to transpose when epigenetic regulation is impaired) may have been activated in these accessions along with *Pong*. However, the six accessions harbored average copy numbers of nine potentially active TEs (Supplementary Table 4). Taken together these data suggest that these six accessions have accumulated silenced *Pong* elements since domestication. Finally, additional evidence for the stability of *Pong* silencing can be inferred from the observation that none of the 2801 accessions lacking *Ping* have a higher *mPing* copy number than accessions with *Ping*.

## Discussion

Results of the evolutionary inventory of the members of the *mPing/Ping/Pong* TE family in wild and domesticated rice genomes suggest the following scenario for the origin of the *mPing* burst. All *mPing* subtypes in domesticated accessions (*mPingA-D*) were generated prior to domestication, probably in *O. rufipogon*, by internal deletion from *Ping16G*. Furthermore, *Ping16G*, but not *Ping16A*, was detected in 21 of 48 *O. rufipogon* accessions. The fact that only 31 of the 3000 extant domesticated accessions examined have *Ping16G* suggests that there has been a massive loss of this element in domesticated rice. In contrast, the *Ping16A* variant was identified in the majority of the domesticated accessions with *Ping* (154/199). Its absence in *O. rufipogon* genomes indicate that it was either very rare in wild populations or that it arose in *japonica* after domestication. *Ping16A* has experienced limited but significant proliferation in *japonica* and has even been introgressed into a small number of *indica* accessions (Table 1). Taken together, these data indicate that *Ping16A* has become more widely distributed in domesticated accessions, whereas *Ping16G* is disappearing.

Yeast assays testing the functional impact of several mutations in and adjacent to the *Ping* TIR demonstrate that the + 16G (*Ping16G*) to + 16A (*Ping16A*) polymorphism significantly reduces transposition frequency. Thus, *Ping16A* encoded proteins (which are identical to *Ping16G* encoded proteins) are more likely to catalyze the transposition of *mPing* (with its + 16G) than *Ping16A*. This situation is reminiscent of other autonomous elements that harbor sequences that reduce transposition frequency^26,27^. It has been hypothesized that autonomous TEs enhance their survival by evolving self-regulating mutations that reduce both host impact and epigenetic detection and silencing^27^.

The vast majority of accessions with *Ping16A* have only one *Ping* (105/154 accessions) and a moderate number of *mPing* elements (mean = 28). One of these accessions is the reference accession Nipponbare where the inability to detect transposition of *Ping* or *mPing* was initially attributed to *Ping* silencing^28^. In fact, *Ping* is not silenced in Nipponbare nor in any other accession analyzed to date^7^. Rather it is transcribed and catalyzes (infrequent) transposition of *mPing*^6,7^. We speculate that accessions with a single copy of *Ping16A* may be experiencing a balance, perhaps under stabilizing selection, between host survival and the maintenance of an active TE family in the genome.

The hypothesized balance between *Ping16A* and *mPing* elements and the host was perturbed in the subset of temperate *japonica* accessions experiencing *mPing* bursts^7^ and it was suggested that the shared *Ping16A_Stow* locus may have been responsible^7^. Based on the evolutionary inventory presented in this study, it follows that *Ping16A_Stow* was generated in a temperate *japonica* accession when *Ping16A* transposed into a *Stowaway* element on chromosome 1. The *Stowaway* element (without the *Ping* insertion) was also present at this locus in *O. rufipogon* (Table 1). It is unlikely that this *Stowaway* is active as there are only four family members, each with < 96% sequence identity, in the Nipponbare genome. Here we find that *Ping16A_Stow* is also shared by five of the six accessions with the highest *mPing* copy numbers among the 3000 accessions analyzed (Table 2). The sixth accession, IRIS_313_15904, has a region of introgressed *indica* or *aromatic* alleles at this location, which may have replaced the *Ping16A_Stow* locus in prior generations. The association of *Ping16A_Stow* with higher *mPing* copy numbers is consistent with its suggested role in triggering *mPing* bursts. However, the mechanism by which *Ping16A_Stow* may initiate the burst is unknown and warrants further investigation. Prior studies indicated that increased *Ping* transcripts were correlated with more *mPing* transpositions in accessions undergoing *mPing* bursts^7,28^. Our unpublished data suggest that *Ping16A_Stow* does not produce more transcripts compared with other *Ping* elements, suggesting that mechanisms other than an increased transcript level from this locus may be responsible.

In conclusion, the results of this study demonstrate that *mPing* bursts are likely restricted to the past century as none of the thousands of genomes analyzed have as many *mPing* (hundreds) and *Ping* (7–10) elements as the four previously characterized accessions. Further, analysis of the 3000 rice genomes and wild progenitors indicates that two variants of the autonomous *Ping* element, *Ping16A* and the subsequently evolved *Ping16A_Stow* locus appear to be critical for *mPing* hyperactivity. Other studies have shown that domestication can be associated with the loss of epigenetic regulation^29^, which may lead to the activation of TEs. However, our data indicate that *Pong* element copy number has been stably maintained from the wild ancestor through the generation of the thousands of domesticated accessions, suggesting that epigenetic regulation was unaffected. In contrast, *Ping* activity has been sustained during domestication, resulting in the spread and amplification of the *Ping16A* variant and the generation of the *Ping16A_Stow* locus in rice. Yet, the spread of *Ping* activity associated with exceptional *mPing* activity has been very limited in rice, likely due to its high level of self-fertilization.

## Methods

### Dataset

Illumina DNA sequencing reads of 3000 rice accessions were obtained from NCBI SRA project PRJEB6180. The metadata incorporating name and origin of the 3000 rice accessions was extracted from previously published Tables S1A and S1B^20^. The raw reads of 48 *O. rufipogon* accessions were obtained from NCBI SRA under project accession numbers listed in Supplementary Data 2. The metadata associated with the subgroup classification of these 48 *O. rufipogon* accessions was extracted from prior studies^10,30^. The raw reads of *Oryza glaberrima*, *Oryza glumaepatula* (also known as *Oryza glumipatula*), and *Oryza meridionalis* were obtained from NCBI SRA projects accession numbers SRR1712585, SRR1712910, and SRR1712972.

### Population structure and ancestral component analysis

The genotyped SNP dataset (release 1.0 3 K RG 4.8 million filtered SNP Dataset) of the 3000 rice genomes was obtained from SNP-Seek Database^31^ (http://snp-seek.irri.org). A subset of 270,329 SNPs was selected by removing SNPs in approximate linkage equilibrium using plink v1.09 (--indep-pairwise 1000 kb 20 kb 0.8)^32^. Population clustering analysis was performed by ADMIXTURE v1.3.0^33^ (-s 2) with *K* from 2 to 10. Most rice accessions clustered into five subgroups (*indica*: IND, *aus*/*boro*: AUS, *aromatic* (*basmati*/*sadri*): ARO, temperate *japonica*: TEJ, and tropical *japonica*: TRJ) when *K* is 5. Using the ancestral analysis of ADMIXTURE under the *K* = 5 model, a rice accession was assigned to one of these five subgroups if it had ≥ 80% of its ancestral component from a given subgroup. Any accessions that had no major ancestral component ( < 80%) were categorized as admixed (ADM) accessions. During the preparation of this study, Wang et al. published an analysis of the same dataset^14^. The subgroup classifications were compared between the two studies and the results are consistent except that Wang et al. identified additional subgroups in *indica* and *japonica*.

The 4.8 million filtered SNPs were imputed and phased with BEAGLE v5.0^34^ using default parameters (impute = true imp-states = 1600 imp-segment = 6.0 cluster = 0.005). A total of 768 accessions with major ancestral component over 99.99% were used as reference panels for five rice subgroups (344 *indica* accessions, 111 *aus/boro* accessions, 31 *aromatic* accessions, 124 temperate *japonica* accessions, and 158 tropical *japonica* accessions). Local ancestry assignment was performed on accessions of interest with RFMix v2.03^35^ using default parameters. Regions of interest were manually inspected in the results of RFMix. Introgression was defined as an allele that is present in one subgroup but has originated from another subgroup.

### Copy numbers characterization

The *mPing*, *Ping*, and *Pong* insertion sites across the 3000 rice genomes were characterized with RelocaTE2 (aligner = BLAT mismatch = 1 len_cut_trim = 10)^19^ using raw reads obtained from NCBI SRA. Element-specific sequence differences were identified and used to distinguish *Ping* and *Pong* from *mPing* insertions (Fig. 1a). Three separate runs of RelocaTE2 were performed using *mPing*, *Ping*, and *Pong* as queries. Paired-end reads where one read of a pair matched the internal sequence of a *Ping* element (253–5164 bp) and the mate matched to a unique genomic region of the Nipponbare reference genome (MSU7) were used to differentiate *Ping* insertions. Similarly, paired-end reads where one read matched the internal *Pong* element sequence (23–5320 bp) and the mate matched to a unique genomic region of MSU7 were used to identify *Pong* insertions. An equivalent approach was undertaken with *mPing* sequences but the prior identified *Ping* and *Pong* insertion sites were removed from the *mPing* RelocaTE2 results to generate final *mPing* insertions. RelocaTE2 analysis was performed in 48 *O. rufipogon* genomes to identify *mPing*, *Ping*, and *Pong* insertions. However, the short insert size and insufficient read depth of *O. rufipogon* sequencing libraries prevented distinguishing *Ping* and *Pong* insertions from *mPing*.

Copy numbers of *mPing*, *Ping*, and *Pong* elements were estimated from the ratio of the element read depth to the genome-wide average read depth using the script “Rice3k_copy_number_depth_window_mPing/Ping.py”. The genome average sequence coverage for each genome was calculated using qualimap v2.1.2^36^. The element read depth was calculated using a window-based approach to capture read depth variation across the element. All sequencing reads associated with a given repeat element were extracted from the RelocaTE2 results. The reads were aligned to the element using BWA v0.7.12^37^ with default parameters (mem -k 19 -w 100 -T 30). Alignments with ≤ 2 mismatches were retained for further analysis. The sequence coverage of each position in the element was calculated using mpileup command in SAMtools v0.1.19^38^ (mpileup -d 8000). Positions 1–430 bp of *mPing* element was divided into 50-bp windows with 40-bp of overlapping sequence between adjacent windows. The read depth of each 50-bp window of *mPing* was extracted from mpileup results. The copy number of each 50-bp window was defined as the ratio of the depth of each 50-bp window to the genome-wide average read depth. Approximate estimation of *mPing* copy numbers was from an average copy number of all 50-bp windows. *Ping* and *Pong* copy numbers were calculated using positions 260–3260 bp so that unique regions in the targeted element were considered for the assessment. To confirm the statistical differences a one-sample *t*-test was performed to determine whether the average read depth of 50-bp windows of a given element was equal to genome-wide average read depth.

The read depth method was evaluated using simulated datasets. Simulated TE insertions were generated by randomly inserting *mPing* elements into rice chromosome 3 (OsChr3) and chromosome 4 (OsChr4) using custom scripts. Copy numbers of 1, 10, 100, and 1000 *mPings* were simulated to evaluate the performance of the read depth on TE copy numbers. Three replicates were generated for each dataset. Sequencing datasets were simulated with pIRS^39^ at varying depths of 1, 2, 3, 4, 5, 10, 20, and 40 to evaluate the performance of the read depth method on sequencing depths. Sequencing reads were aligned to OsChr3 and OsChr4 with SpeedSeq^40^ (align -t 24 -R “@RG/tID:id/tSM:sample/tLB:library”), which uses BWA (mem -k 19 -w 100 -T 30) to align reads, Sambamba^41^ (-M 20) to sort alignments, and SAMBLASTER^42^ (-c 2 -m 20) to mark PCR duplicates. Genome-wide sequencing depths were obtained with qualimap using BAM files generated by SpeedSeq. *mPing*-related reads were obtained with RelocaTE2 (--size 500 --mismatch 2 --aligner blat) and were aligned to *mPing* sequence with BWA (mem -k 19 -w 100 -T 30). Alignments with ≤ 2 mismatches were retained for further analysis. The sequence coverage of each position in the element was calculated using mpileup command in SAMtools (mpileup -d 100000). *mPing* copy numbers were characterized with the script “Rice3k_copy_number_depth_window_mPing.py”. The results indicate that the read depth method can estimate approximate *mPing* copy numbers with a wide range of sequencing depth (Supplementary Figure 7). Even at a low coverage of 2 where RelocaTE2 shows low efficiency to identify TE insertions, the read depth method can accurately estimate *mPing* copy number when there is only a single element in the genome (Supplementary Figure 7a).

The presence and absence of *mPing*, *Ping*, and *Pong* were also confirmed with manual inspection. Briefly, *mPing*, *Ping*, and *Pong*-associated reads were extracted and aligned to the elements as described above. The sequence coverage of *mPing*, *Ping*, and *Pong* were inspected using heatmap and Integrative Genomics Viewer (IGV) v2.3.0^43^. Only accessions showing sequence coverage across a given element (generally needs ≥ 70% of elements covered) were defined as accessions having this element. This approach was also used to identify four *Aus*/*boro* accessions that have a *Ping* locus (Chr11: 25822230–25802232) that was not identified with RelocaTE2.

### Analysis of *Ping16A_Stow*

The pre-aligned BAM files of 3000 rice genomes (http://s3.amazonaws.com/3kricegenome/Nipponbare/”Accession_Name”.realigned.bam) were analyzed to determine if a *Stowaway* element was present at the *Ping16A_Stow* locus Chr1: 2640500–2640502. A total of 199 rice genomes with signatures of TE insertions at the *Ping16A_Stow* locus (reads with only partial “soft clipped” alignments) were analyzed to confirm the *Stowaway* insertion. A pseudogenome was built of a single *Stowaway* element and its 2-kb flanking sequences at position Chr1: 2640500–2640502. The sequencing reads from each of the 199 rice genomes were aligned to the pseudogenome using BWA with default parameters (mem -k 19 -w 100 -T 30) followed by analysis of the BAM files to identify junction reads covering both the *Stowaway* and its flanking sequence. All of these 199 accessions were confirmed to have the *Stowaway* element at position Chr1: 2640500–2640502.

A similar approach that identified the *Stowaway* insertion was used to identify *Ping* insertions in the *Stowaway* element at the *Ping16A_Stow* locus. A pseudogenome was built using a *Ping* element and its flanking sequences, which are 1–305 bp of the *Stowaway* element upstream of *Ping* and 306–770 bp of the *Stowaway* element downstream of *Ping*. The sequencing reads of these 199 rice genomes were aligned to the pseudogenome using BWA with default parameters (mem -k 19 -w 100 -T 30). Analysis of junction reads covering both *Ping* element and its flanking *Stowaway* element identified eleven accessions having a *Ping* insertion in the *Stowaway* element at the *Ping16A_Stow* locus (Supplementary Table 3).

### Analysis of + 16 G/A SNP genotype

A locus-specific approach was used to analyze the genotype of the + 16 G/A SNP of the *Ping* element in rice. *Ping*-containing reads of each locus were extracted from the RelocaTE2 results and the reads were aligned to the Nipponbare *Ping* element using BWA with default parameters (mem -k 19 -w 100 -T 30). Alignments with ≤ 2 mismatches were analyzed using mpileup command in SAMtools (mpileup –d 8000) to generate a read depth profile, which includes base composition information at each position. The nucleotide counts at the + 16 G/A SNP were obtained from the read depth profile. A *Ping* with two or more reads supporting G was genotyped as *Ping16G*, whereas a *Ping* locus with two or more reads supporting A was genotyped as *Ping16A*. The genotypes of three *Ping* loci, including Chr6: 23521641–23526981 (Nipponbare *Ping*), Chr1: 264050–2640502 (*Ping16A_Stow*), and Chr11: 25822230–25802232 (a *Ping* locus in *Aus*/*boro*), were assigned through manual inspection because these loci were either reference *Ping* (53 accessions with Nipponbare *Ping*) or nonreference *Ping* but have not been identified with RelocaTE2 (11 accessions with *Ping16A_Stow* and 4 accessions with the *Aus*/*boro Ping* locus).

For *O. rufipogon*, all reads aligning to *mPing*, *Ping*, and *Pong* were pooled to analyze the base composition at the + 16 G/A SNP because *mPing*, *Ping*, and *Pong* insertions could not be efficiently sorted. An *O. rufipogon* genome was categorized as a genome having *Ping16G* or *Ping16A* based on whether they had two or more reads supporting G or A. Accessions that have two or more reads supporting both G and A were further analyzed to clarify whether the *Ping16A* is present in these genomes. For example, accession W1230 had both G (288 reads) and A (23 reads) at the + 16G/A SNP. These A-supporting reads and their mates were extracted from W1230 sequences and aligned to pseudogenomes that have W1230 *mPing* or *Ping* inserted in MSU7. All of these A-supporting reads were uniquely aligned to *mPing* locus Chr3: 25526483–25526485 that contains a 430-bp *mPingC* element successfully assembled from locus-specific paired-end reads, suggesting these A-supporting reads were from *mPing* not from *Ping*.

### Assembly and classification of *mPing* sequences

A locus-specific assembly was performed to recover full-length *mPing* sequences from rice sequences. The sequencing reads matching *mPing* were obtained using RelocaTE2, assembled using velvet v1.2.09 (MAXKMERLENGTH = 31 -ins_length 500 -exp_cov auto -scaffolding yes)^44^. The flanking non-*mPing* sequences were removed from the assembled sequences. Any *mPing* candidate loci containing sequence gaps were removed from the analysis. The remaining full-length *mPing* sequences were compared using BLAST v2.2.26 to build an undirected graph with python package NetworkX (https://networkx.github.io). Each node in the graph is an *mPing* sequence and each edge is a connection, which requires two *mPing* sequences are properly aligned (number of gaps or mismatches ≤ 4). The *mPing* sequences in each subgraph represent a subtype of *mPing*. Representative sequences were extracted from each *mPing* subtype and aligned with four canonical defined *mPing* subtypes (*mPingA*, *mPingB*, *mPingC*, and *mPingD*) from the prior study^5^ using MUSCLE v3.8.425^45^ with default parameters (-maxiters 16). The multiple sequence alignment in MSA format was converted into VCF format using msa2vcf.jar tool (https://github.com/lindenb/jvarkit) to identify polymorphic sites. The assembled *mPing* sequences were classified into subtypes based on their breakpoints and point mutations compared with the four canonical *mPing* subtypes.

The reads of *O. rufipogon* accessions were aligned to four canonical defined *mPing* subtypes (*mPingA*, *mPingB*, *mPingC*, and *mPingD*) using BWA with default parameters (mem -k 19 -w 100 -T 30). Alignments with ≤ 2 mismatches were manually inspected using IGV v2.3.0 to determine if the reads cover breakpoint of each *mPing* subtype in each accession. An accession with two or more reads covering the breakpoint of an *mPing* subtype was identified as an accession containing this *mPing* subtype.

### Phylogenetic analysis

The 270,329 SNPs used for ADMIXTURE analysis were used to genotype HEG4, EG4, A119, and A123 using GATK UnifiedGenotyper v3.4-46^46^. The phylogenetic tree of rice accessions was built using a neighbor-Joining method implemented in FastTree v2.1.10 (-noml -nome)^47^. The sequencing reads for the 48 *O. rufipogon* accessions were analyzed to obtain a SNP dataset. Briefly, paired-end reads were aligned to MSU7 using SpeedSeq v 0.1.0 (align -t 24 -R “@RG/tID:id/tSM:sample/tLB:library”). The resulting BAM files were analyzed with GATK UnifiedGenotyper to perform SNP calling. Filtering parameters (QD < 2.0, MQ < 40.0, FS > 60.0, AF < 0.05, HaplotypeScore > 13.0, MQRankSum < –12.5, ReadPosRankSum < –8.0, MQ0 > = 4 && ((MQ0/(1.0×DP)) > 0.1), QUAL < 30.0, DP < 6, DP > 5000, HRun > 5) were used to retain high-quality SNPs using GATK VariantFiltration. Only homozygous SNPs that did not overlap the repetitive sequences were used in the phylogenetic analysis. These high-quality SNPs were extracted and converted into PHYLIP format multiple sequence alignment for phylogenetic analysis with RAXML v8.2.8^48^ under a GTRGAMMA model (-m GTRGAMMA). Bootstrap was performed using 100 iterations (-f a -# 100). *O. glaberrima*, *Oryza glumaepatula*, and *O. meridionalis* were treated as outgroups. Graphical representations of the phylogenetic trees were generated in R using “APE” libraries^49^.

### Yeast transposition assay

*mPing* was amplified with Phusion High-Fidelity PCR Master Mix (Thermo Fisher Scientific) using the control *mPing* primers (*mPing* F and *mPing* R) or mutation containing primers (i.e., *mPing* F and *mPing16A* R; Supplementary Table 5). The primary PCR products were then amplified with *ADE2* TSD F and *ADE2* TSD R primers (Supplementary Table 5) to add *ADE2* homologous sequences. Purified PCR products were co-transformed into *Saccharomyces cerevisiae* strain JIM17^50^ with *Hpa*I digested pWL89a plasmid using the lithium acetate/polyethylene glycol method^51^. Plasmids were isolated from individual yeast clones using the Zymo Yeast Plasmid Miniprep kit (Zymo Research) and transformed into *Escherichia coli* for plasmid purification and sequence validation.

Sequence verified plasmids were transformed into *S. cerevisiae* strain CB101^50^ containing previously described pAG413 GAL *ORF1* Shuffle1 NLS and pAG415 GAL *Pong TPase* L384A, L386A plasmids^25^. The transposition rate was measured as described in the prior study^18^. Briefly, 3 ml cultures were grown in CSM-His-Leu-Ura (dextrose) for 24 h at 30 °C, and 100 µl was plated onto 100 mm CSM-His-Leu-Ura-Ade (galactose) plates. The total number of yeast cells was calculated by plating a 10^-4^ dilution of the cultures onto YPD plates. The numbers of colonies on the galactose plates were determined after 10 days of incubation at 30 °C. The transposition rate was determined by dividing the galactose colony count by the total number of cells plated.

### Statistical analysis

Sample sizes, statistical tests, and *P-*values are indicated in figures or figure legends. Linear regression, two-tailed Pearson’s correlation, two-tailed Wilcoxon–Mann–Whitney, one-way ANOVA and Tukey’s HSD test were performed with “lm”, “cor.test”, “wilcox.test”, “aov”, and “TukeyHSD” functions in R. One-sample *t*-test was performed with ‘ttest_1sample’ function in Python module ‘sci.stats’.

### Code availability

RelocaTE2 and other code used in this study are available at https://github.com/stajichlab/Dynamic_rice_publications or 10.5281/zenodo.1492794.

## Supplementary information

Supplementary Information

Peer Review

Description of Additional Supplementary Data

Supplementary Data 1

Supplementary Data 2

Source Data

Reporting summary

## Data Availability

A reporting summary for this article is available as a Supplementary Information file. Illumina DNA sequencing reads have been obtained from NCBI SRA project PRJEB6180, SRR1712585, SRR1712910, and SRR1712972. SNPs and BAM files have been obtained from 3000 Rice Genomes Project On AWS [https://registry.opendata.aws/3kricegenome/]. Source data for Figs. 1b–g, Figs. 2a, b, d, e, Fig. 4b, Supplementary Figure 2-3, and Supplementary Figure 6 are provided in Supplementary Data 1-2. Source data for Fig. 3b and Supplementary Figure 5 are provided as a Source Data file. Yeast strains used in this study are readily available from C. Nathan Hancock lab upon request (NathanH@usca.edu).
