# Supplementary material for: Tracking the origin of two genetic components associated with transposable element bursts in domesticated rice
Source: Nat Commun. 2019 Feb 7;10:641. doi: 10.1038/s41467-019-08451-3 (PMC6367367; doi:10.1038/s41467-019-08451-3)
Supplement: Supplementary file 1 — Supplementary Information [file 41467_2019_8451_MOESM1_ESM.docx]

**Tracking the origin of two genetic components associated with transposable element bursts in domesticated rice**

Chen *et al.*


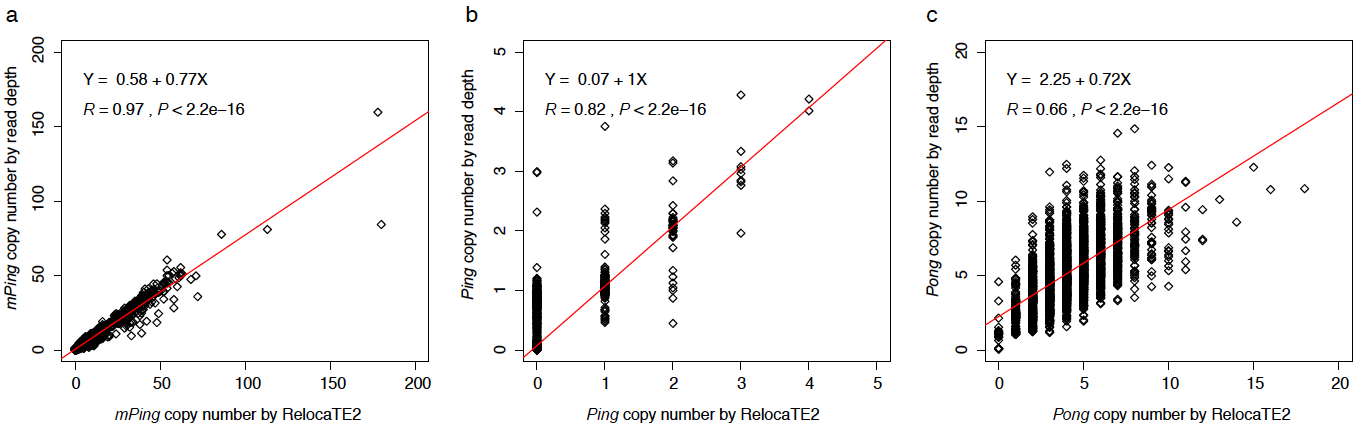


**Supplementary Figure 1. Comparison of *mPing*, *Ping* and *Pong* copy number estimates in 3,000 rice accessions using the RelocaTE2 and read-depth methods.** **a**, *mPing*. **b**, *Ping*. **c**, *Pong*. The results of RelocaTE2 and read-depth methods were modeled with linear regression using R. Regression lines are shown in red. Statistical significance of linear relationship was tested by a two-tailed Pearson’s correlation test.


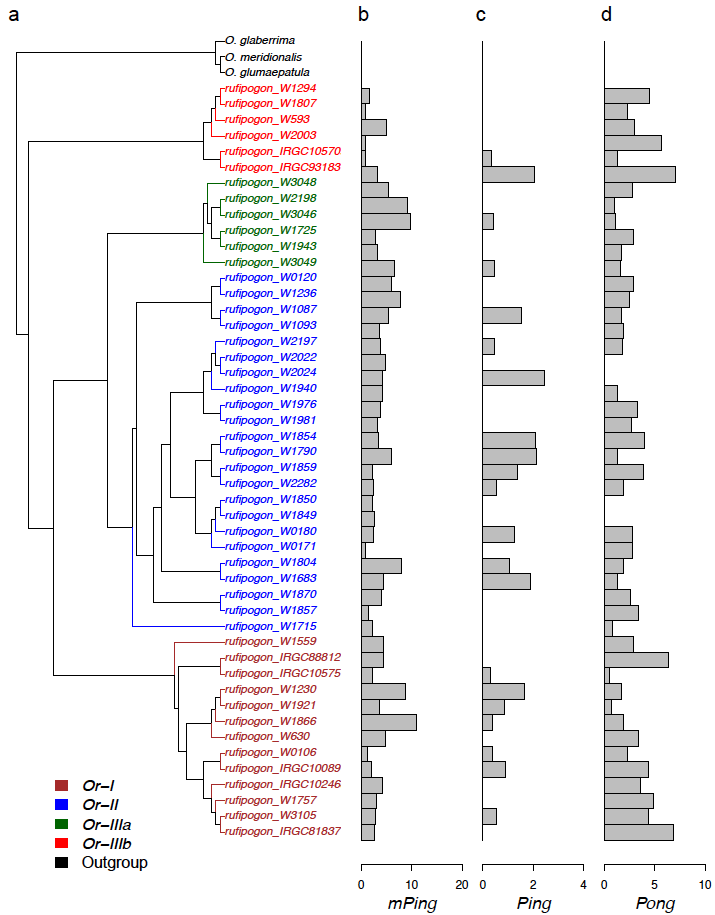


**Supplementary Figure 2. Copy numbers of *mPing*, *Ping* and *Pong* elements in the sequenced genomes of *O.* *rufipogon*.** **a**, Phylogenetic tree of *O. rufipogon*. Details of phylogenetic analysis were described in Methods. **b**, Copy number of *mPing* in *O. rufipogon*. **c**, Copy number of *Ping* in *O. rufipogon*. **d**. Copy number of *Pong* in *O. rufipogon*. Source data for Supplementary Figure 2 are provided in Supplementary Data 2.


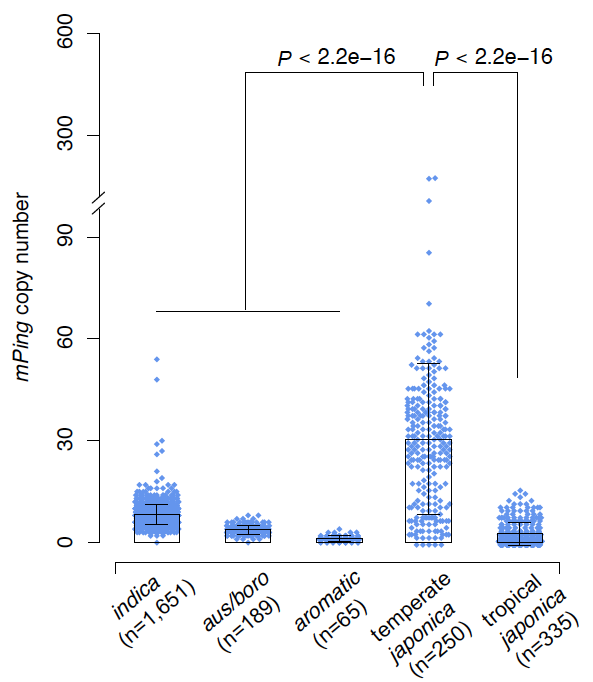


**Supplementary Figure 3. Comparison of *mPing* copy number in different rice subgroups.** Each dot represents a rice accession. The x-axis indicates the accession ecotype. The y-axis indicates *mPing* copy number. Error bars show the standard deviation (s.d.) of each subgroup. Differences in *mPing* copy number among subgroups were tested by a one-way ANOVA (*P* value < 2e-16, *F* value = 613.6, DF = 4) followed by a Tukey’s honest significant difference (Tukey’s HSD) test. Source data for Supplementary Figure 3 are provided Supplementary Data 1.


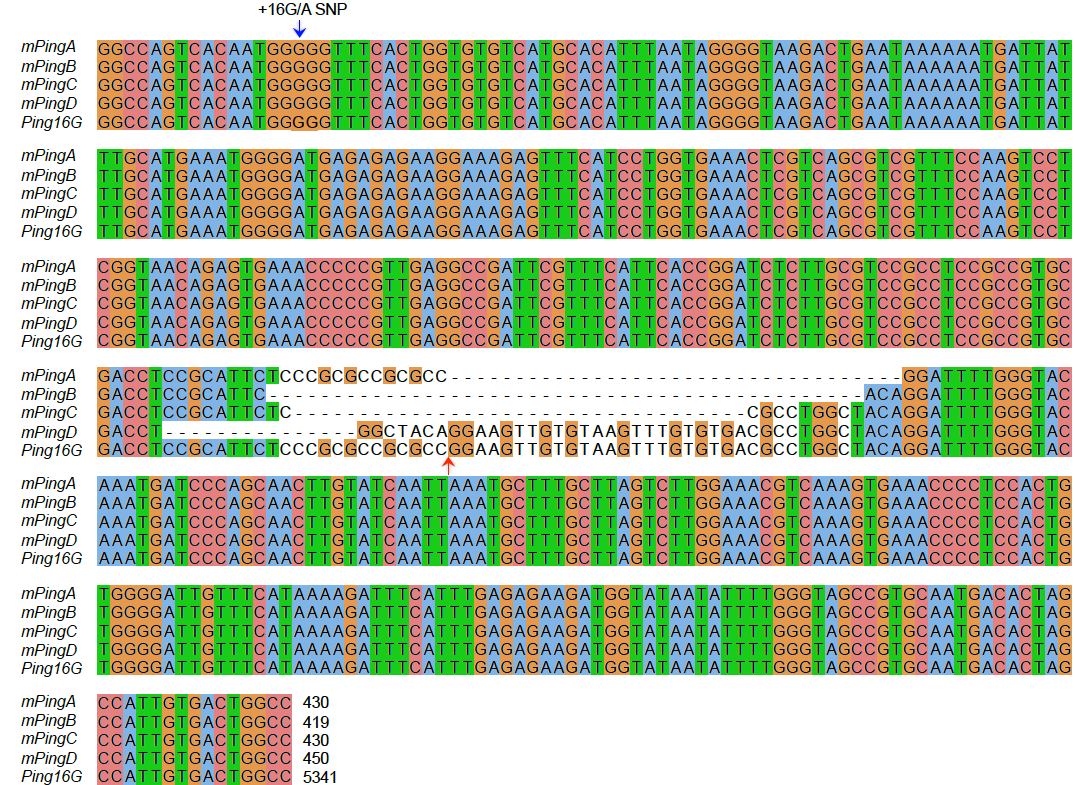


**Supplementary Figure 4. Sequence alignment of *mPing* variants and homologous *Ping* sequences (*Ping16G*).** The blue arrow indicates the position of +16G/A SNP. The red arrow indicates the breakpoint in *Ping*, with the internal sequence not shared with *mPing* not shown. The number at the end of each sequence indicates the original sequence length of *mPing* or *Ping* element.


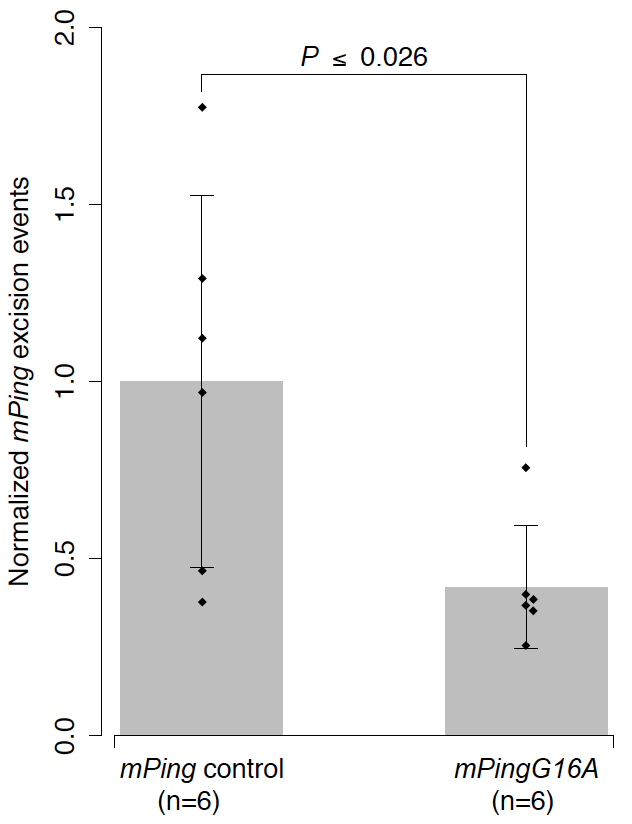


**Supplementary Figure 5. Transposition frequency of *mPing* and *mPingG16A* using the *Ping* *ORF1* and *TPase* proteins.** The x-axis indicates *mPing* variants. The y-axis shows *mPing* transposition frequency measured as excision events per million cells and normalized to the control *mPing*. Error bars show the standard deviation (s.d.) of six independent biological replicates. *P* value is based on a two-tailed Wilcoxon-Mann-Whitney test. Source data for Supplementary Figure 5 are provided as a Source Data file.


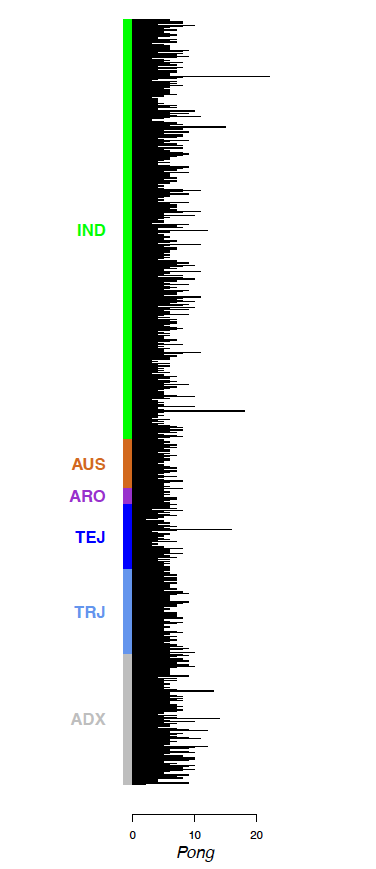


**Supplementary Figure 6. *Pong* copy numbers in 3,000 genomes**. Colors represent the five major rice subgroups: *indica* (IND), *aus/boro* (AUS), *aromatic* (ARO), temperate *japonica* (TEJ), tropical *japonica* (TRJ), and admixed (ADM). X axis indicates *Pong* copy numbers that were estimated by RelocaTE2. Source data for Supplementary Figure 6 are provided Supplementary Data 1.


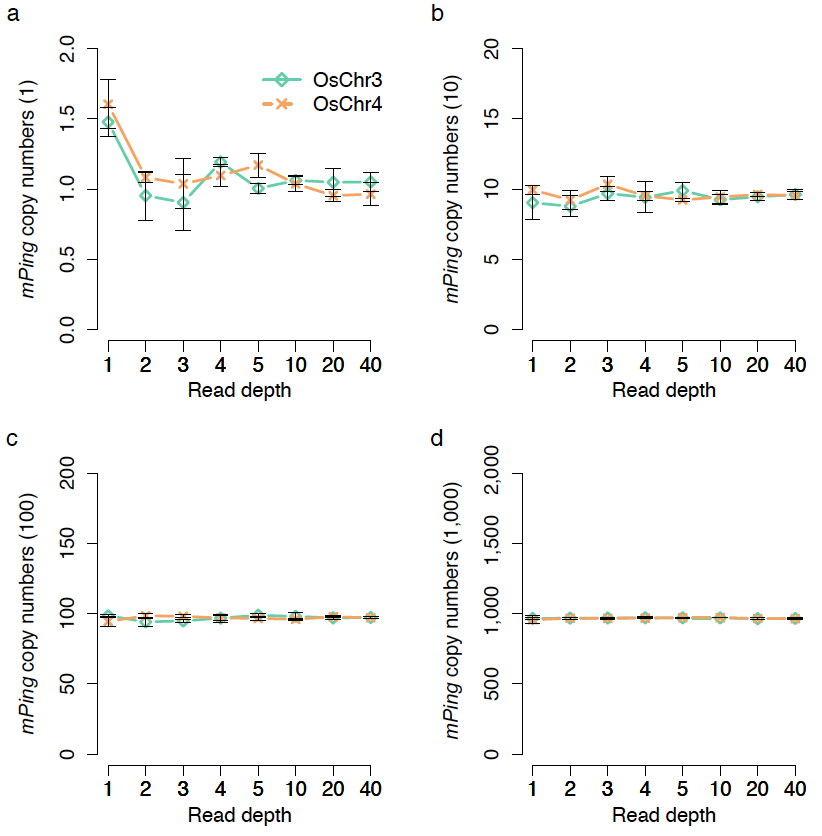


**Supplementary Figure 7. Evaluation of copy number estimation with a read-depth method using simulated data.** Random *mPing* insertions were simulated for OsChr3 and OsChr4 with copy numbers of 1 (**a**), 10 (**b**), 100 (**c**), and 1,000 (**d**). Three replicates were generated for each parameter combination (*mPing* copy numbers and chromosomes). Sequencing datasets were simulated with pIRS at varying depths of 1, 2, 3, 4, 5, 10, 20, and 40. X axis indicates sequencing depth or read depth. Y axis indicates estimated *mPing* copy numbers using the read-depth method. The error bars show the standard deviation (s.d.) among three replicates.

**Supplementary Table 1. Copy numbers of *mPing* in domesticated rice and *O. rufipogon***

| Subgroups | Number of accessions | Average *mPing* copy number | Range of *mPing* copy number |
| --- | --- | --- | --- |
| *O. sativa* | 3,000 | 9.1 | 0-180 |
| *-indica* | 1,651 | 8.2 | 0-54 |
| *-aus/boro* | 189 | 3.8 | 0-8 |
| *-*temperate *japonica* | 250 | 30.5 | 0-180 |
| *-*tropical *japonica* | 335 | 2.6 | 0-16 |
| *-aromatic* | 65 | 1.2 | 0-4 |
| *-*admixed | 510 | 9.3 | 0-72 |
| *O. rufipogon* | 48 | 4.0 | 0.7-10.9 |
| *-Or-I* | 13 | 4.2 | 1.2-10.9 |
| *-Or-II* | 23 | 3.9 | 0.8-7.9 |
| *-Or-IIIa* | 6 | 6.1 | 2.8-9.7 |
| *-Or-IIIb* | 6 | 2.1 | 0.7-5.0 |
|  |  |  |  |

**Supplementary Table 2. Distribution of distinct *mPing* types**

| Subgroups | Number of accessions | *mPingA* | *mPingB* | *mPingC* | *mPingD* |
| --- | --- | --- | --- | --- | --- |
| *O. sativa* | 3,000 | 1,476 | 1,746 | 653 | 234 |
| *-indica* | 1,651 | 981 | 1,255 | 442 | 116 |
| *-aus/boro* | 189 | 6 | 68 | 56 | 78 |
| *-*temperate *japonica* | 250 | 209 | 128 | 1 | 0 |
| *-*tropical *japonica* | 335 | 20 | 83 | 81 | 0 |
| *-aromatic* | 65 | 20 | 10 | 2 | 1 |
| *-*admixed | 510 | 240 | 202 | 71 | 39 |
| *O. rufipogon* | 48 | 24 | 29 | 33 | 16 |
| *-Or-I* | 13 | 3 | 10 | 11 | 8 |
| *-Or-II* | 23 | 10 | 11 | 19 | 7 |
| *-Or-IIIa* | 6 | 6 | 5 | 2 | 1 |
| *-Or-IIIb* | 6 | 5 | 3 | 1 | 0 |

**Supplementary Table 3. *Ping* and *mPing* copy numbers in accessions with *Ping16A_Stow***

| Name | *Ping* copy number | *mPing* copy number | Subgroup |
| --- | --- | --- | --- |
| B160 | 3 | 180 | temperate *japonica* |
| B235 | 2 | 113 | temperate *japonica* |
| B005 | 1 | 86 | temperate *japonica* |
| B003 | 2 | 72 | admixed |
| B001 | 2 | 71 | temperate *japonica* |
| IRIS_313-11655 | 3 | 68 | admixed |
| B168 | 1 | 54 | admixed |
| IRIS_313-10839 | 1 | 37 | temperate *japonica* |
| CX212 | 1 | 37 | temperate *japonica* |
| IRIS_313-11202 | 2 | 32 | temperate *japonica* |
| IRIS_313-10124 | 1 | 29 | temperate *japonica* |

**Supplementary Table 4. Copy numbers of TEs in selected rice accessions with high copy numbers of *Pong***

| Accessions | Subgroup | *Pong* | *mPing* | *nDart* | *Gaijin* | *spmlike* | *Truncator* | *mGing* | *Dasheng* | *Retro1* | *RIRE2* | *RIRE3* |
| --- | --- | --- | --- | --- | --- | --- | --- | --- | --- | --- | --- | --- |
| Mean  Max | - | 4  25 | 9  180 | 5  30 | 34  92 | 89  211 | 13  34 | 52 119 | 16  40 | 7.7  21 | 83  170 | 133  276 |
| B213 | *indica* | 18 | 8 | 5 | 19 | 76 | 17 | 28 | 17 | 9 | 84 | 117 |
| IRIS_313-11598 | admixed | 14 | 12 | 4 | 44 | 118 | 19 | 58 | 25 | 11 | 155 | 144 |
| IRIS_313-12003 | temperate *japonica* | 16 | 25 | 3 | 32 | 41 | 0 | 61 | 7 | 6 | 33 | 89 |
| IRIS_313-12133 | *indica* | 15 | 8 | 7 | 26 | 90 | 14 | 43 | 14 | 9 | 94 | 128 |
| IRIS_313-8703 | *indica* | 25 | 9 | 15 | 52 | 150 | 20 | 63 | 14 | 9 | 101 | 184 |

**Supplementary Table 5. Primer sequences used in this study**

| Primer names | Sequence |
| --- | --- |
| *mPing* F | 5’ – CATGATTGTGAGGTCTGTTAGGCCAGTCACAATGGCTAGTGTC – 3’ |
| *mPing* R | 5’ – GTAAGAAAACACTAAACCGTTAAGGCCAGTCACAATGGGGGTTTC – 3’ |
| *mPing1T* R | 5’ – GTAAGAAAACACTAAACCGTTAA**T**GCCAGTCACAATGGGGGTTTC – 3’ |
| *mPing2T* R | 5’ – GTAAGAAAACACTAAACCGTTAAG**T**CCAGTCACAATGGGGGTTTC – 3’ |
| *mPing3A* R | 5’ – GTAAGAAAACACTAAACCGTTAAGG**A**CAGTCACAATGGGGGTTTC – 3’ |
| *mPing4A* R | 5’ – GTAAGAAAACACTAAACCGTTAAGGC**A**AGTCACAATGGGGGTTTC – 3’ |
| *mPing5C* R | 5’ – GTAAGAAAACACTAAACCGTTAAGGCC**C**GTCACAATGGGGGTTTC – 3’ |
| *mPing6T* R | 5’ – GTAAGAAAACACTAAACCGTTAAGGCCA**T**TCACAATGGGGGTTTC – 3’ |
| *mPing7G* R | 5’ – GTAAGAAAACACTAAACCGTTAAGGCCAG**G**CACAATGGGGGTTTC – 3’ |
| *mPing8A* R | 5’ – GTAAGAAAACACTAAACCGTTAAGGCCAGT**A**ACAATGGGGGTTTC – 3’ |
| *mPing9C* R | 5’ – GTAAGAAAACACTAAACCGTTAAGGCCAGTC**C**CAATGGGGGTTTC – 3’ |
| *mPing10A* R | 5’ – GTAAGAAAACACTAAACCGTTAAGGCCAGTCA**A**AATGGGGGTTTC – 3’ |
| *mPing11C* R | 5’ – GTAAGAAAACACTAAACCGTTAAGGCCAGTCAC**C**ATGGGGGTTTC – 3’ |
| *mPing12C* R | 5’ – GTAAGAAAACACTAAACCGTTAAGGCCAGTCACA**C**TGGGGGTTTC – 3’ |
| *mPing13G* R | 5’ – GTAAGAAAACACTAAACCGTTAAGGCCAGTCACAA**G**GGGGGTTTC – 3’ |
| *mPing14T* R | 5’ – GTAAGAAAACACTAAACCGTTAAGGCCAGTCACAAT**T**GGGGTTTC – 3’ |
| *mPing15T* R | 5’ – GTAAGAAAACACTAAACCGTTAAGGCCAGTCACAATG**T**GGGTTTC – 3’ |
| *mPing16A* R | 5’ – GTAAGAAAACACTAAACCGTTAAGGCCAGTCACAATGG**A**GGTTTC – 3’ |
| *mPing17T* R | 5’ – GTAAGAAAACACTAAACCGTTAAGGCCAGTCACAATGGG**T**GTTTC – 3’ |
| *ADE2* TSD F | 5’ – ACTAAAGAATTAGCAGTCATGATTGTGAGATCTGTTA – 3’ |
| *ADE2* TSD R | 5’ – AGTCTCTACAATTGGGTAAGAAAACACTAAACCGTTAA – 3’ |
