# Supplementary material for: Tracking the origin of two genetic components associated with transposable element bursts in domesticated rice
Source: Nat Commun. 2019 Feb 7;10:641. doi: 10.1038/s41467-019-08451-3 (PMC6367367; doi:10.1038/s41467-019-08451-3)
Supplement: Supplementary file 3 — Description of Additional Supplementary Data [file 41467_2019_8451_MOESM3_ESM.pdf]

Description of Additional Supplementary Files

File Name: Supplementary Data 1
Description: *O. sativa* genomes and estimated copy numbers of *mPing, Ping,* and *Pong*

File Name: Supplementary Data 2
Description: *O. rufipogon* genomes and estimated copy numbers of *mPing, Ping,* and *Pong*

File Name: Source Data for Figure 3b
Description: Transposition frequency of *mPing* variants with mutations at the 5' end in the yeast assay

File Name: Source Data for Supplementary Figure 5
Description: Transposition frequency of *mPing* and *mPingG16A* using the *Ping ORF1* and *TPase* proteins
