# Supplementary material for: Tracking the origin of two genetic components associated with transposable element bursts in domesticated rice
Source: Nat Commun. 2019 Feb 7;10:641. doi: 10.1038/s41467-019-08451-3 (PMC6367367; doi:10.1038/s41467-019-08451-3)
Supplement: Supplementary file 7 — Reporting summary [file 41467_2019_8451_MOESM7_ESM.pdf]

nature research

Corresponding author(s): Susan R. Wessler

Last updated by author(s): Dec 27, 2018

Reporting Summary

Nature Research wishes to improve the reproducibility of the work that we publish. This form provides structure for consistency and transparency in reporting. For further information on Nature Research policies, see Authors & Referees and the Editorial Policy Checklist.

Statistics

For all statistical analyses, confirm that the following items are present in the figure legend, table legend, main text, or Methods section.

n/a	Confirmed	
☐	☒	The exact sample size (*n*) for each experimental group/condition, given as a discrete number and unit of measurement
☐	☒	A statement on whether measurements were taken from distinct samples or whether the same sample was measured repeatedly
☐	☒	The statistical test(s) used AND whether they are one- or two-sided<br>*Only common tests should be described solely by name; describe more complex techniques in the Methods section.*
☒	☐	A description of all covariates tested
☐	☒	A description of any assumptions or corrections, such as tests of normality and adjustment for multiple comparisons
☐	☒	A full description of the statistical parameters including central tendency (e.g. means) or other basic estimates (e.g. regression coefficient) AND variation (e.g. standard deviation) or associated estimates of uncertainty (e.g. confidence intervals)
☐	☒	For null hypothesis testing, the test statistic (e.g. *F*, *t*, *r*) with confidence intervals, effect sizes, degrees of freedom and *P* value noted<br>*Give P values as exact values whenever suitable.*
☒	☐	For Bayesian analysis, information on the choice of priors and Markov chain Monte Carlo settings
☒	☐	For hierarchical and complex designs, identification of the appropriate level for tests and full reporting of outcomes
☐	☒	Estimates of effect sizes (e.g. Cohen's *d*, Pearson's *r*), indicating how they were calculated

Our web collection on statistics for biologists contains articles on many of the points above.

Software and code

Policy information about availability of computer code

Data collection	The sequence data was collected and published by Wang et al. Details were described in Wang et al (https://doi.org/10.1038/s41586-018-0063-9) and the 3,000 rice genomes project (https://doi.org/10.1186/2047-217X-3-7).
Data analysis	The following softwares or tools were used in this study:<br>population structure: ADMIXTURE v1.3.0; imputation: BEAGLE v5.0; introgression analysis: RFMix v2.03; TE insertions: RelocaTE2 (https://github.com/stajichlab/RelocaTE2) archive of code used in this analysis available at https://doi.org/10.5281/zenodo.1344714; reads mapping: BWA v0.7.12, SpeedSeq v0.1.0; SAM/BAM format: SAMtools v0.1.19; SNP calling: GATK UnifiedGenotyper v3.4-46; genome coverage: qualimap v2.1.2; homologous search: BLAST v2.2.26; undirected graph: NetworkX (https://networkx.github.io); multiple sequence alignment: MUSCLE v3.8.425; VCF format: msa2vcf.jar (https://github.com/lindenb/jvarkit); read alignment viewer: IGV v2.3.0; phylogenetic analysis: FastTree v2.1.10, RAXML v8.2.8.<br><br>The custom code that was used in this study is available in the github repositorty https://github.com/stajichlab/Dynamic_rice_publications and archived under DOI https://doi.org/10.5281/zenodo.1492794.

For manuscripts utilizing custom algorithms or software that are central to the research but not yet described in published literature, software must be made available to editors/reviewers. We strongly encourage code deposition in a community repository (e.g. GitHub). See the Nature Research guidelines for submitting code & software for further information.

Data

Policy information about availability of data

All manuscripts must include a data availability statement. This statement should provide the following information, where applicable:

- Accession codes, unique identifiers, or web links for publicly available datasets
- A list of figures that have associated raw data
- A description of any restrictions on data availability

Data Availability

A reporting summary for this Article is available as a Supplementary Information file. Illumina DNA sequencing reads have been obtained from NCBI SRA project PRJEB6180 (https://www.ncbi.nlm.nih.gov/sra/?term=PRJEB6180), SRR1712585 (https://www.ncbi.nlm.nih.gov/sra/?term=SRR1712585), SRR1712910 (https://www.ncbi.nlm.nih.gov/sra/?term=SRR1712910), and SRR1712972 (https://www.ncbi.nlm.nih.gov/sra/?term=SRR1712972). SNPs and BAM files have been obtained from 3000 Rice Genomes Project On AWS (https://registry.opendata.aws/3kricegenome/). Source data for Figs. 1b-g, Figs. 2a,b,d,e, Fig. 4b, Supplementary Figure 2-3, and Supplementary Figure 6 are provided in Supplementary Data 1-2. Source data for Fig. 3b and Supplementary Figure 5 are provided as a Source Data file. Yeast strains used in this study are readily available from Hancock's lab upon request.

Field-specific reporting

Please select the one below that is the best fit for your research. If you are not sure, read the appropriate sections before making your selection.

☒ Life sciences ☐ Behavioural & social sciences ☐ Ecological, evolutionary & environmental sciences

For a reference copy of the document with all sections, see nature.com/documents/nr-reporting-summary-flat.pdf

Life sciences study design

All studies must disclose on these points even when the disclosure is negative.

Sample size	The sample size of the yeast assay is 6-9 to ensure the reproducibility of the experiment. No statistical method or sample-size calculations were used to determine the sample size.
Data exclusions	In Supplementary Figure 3, we excluded the admixed subgroup in the analysis because the origin of this group is obscure. However, the exclusion of this subgroup does not change any conclusion in this study.
Replication	We used a yeast assay to assess the transposition frequency of mutations at terminal inverted repeats of mPing/Ping elements. Each mutation has 6-9 biological replicates. In addition, we tested a key mutation (+16G/A) using both Ping transposases (Figure 3) and Pong transposases (Supplementary Figure 5). These results provide another layer of replicate to support the conclusion.
Randomization	The rice and wild rice strains that were used in this study were classified into different groups based on genetic variations.
Blinding	The raw sequence data was collected and published by other research groups. We were blinded to data collection. We recalculated the group assignment because the group assignment of the 3,000 rice strains has not been published when we started this project years ago. However, we compared the group assignment in this study with results in the publication by Wang at al. 2018. The results are consistent between these two studies. We described the comparison in the method section.

Reporting for specific materials, systems and methods

We require information from authors about some types of materials, experimental systems and methods used in many studies. Here, indicate whether each material, system or method listed is relevant to your study. If you are not sure if a list item applies to your research, read the appropriate section before selecting a response.

Materials & experimental systems

n/a	Involved in the study	
☒	☐	Antibodies
☒	☐	Eukaryotic cell lines
☒	☐	Palaeontology
☒	☐	Animals and other organisms
☒	☐	Human research participants
☒	☐	Clinical data

Methods

n/a	Involved in the study	
☒	☐	ChIP-seq
☒	☐	Flow cytometry
☒	☐	MRI-based neuroimaging
