# Supplementary material for: Tracking the origin of two genetic components associated with transposable element bursts in domesticated rice
Source: Nat Commun. 2019 Feb 7;10:641. doi: 10.1038/s41467-019-08451-3 (PMC6367367; doi:10.1038/s41467-019-08451-3)
Supplement: Supplementary file 2 — Peer Review [file 41467_2019_8451_MOESM2_ESM.pdf]

Journal peer review information: *Nature Communications* thanks Ronald Chalmers, Terence Brown and Francois Sabot for their contribution to the peer review of this work. Peer reviewer reports are available.

Reviewers' comments:

Reviewer #1 (Remarks to the Author):

This is a very significant paper that addresses important questions regarding genome dynamics in rice, which is of particular interest because of its importance in the food economy of the world. Although the results are largely descriptive, they formulate further important questions, which warrant further investigation. Of course, important insights rely on posing good questions, which this work certainly does.

The authors previously published a paper (Lu, l et al, PNAS, 2017) where they characterized fours rice strains undergoing a burst of a transposable element (TE), mPing. They now analyse the genome of ~3,000 strains of domesticated and wild rice to investigate if other mechanisms could regulate the mPing burst. They analyzed the distribution of the mPing/Ping/Pong TE family in the different strains and found that a single nucleotide polymorphism (SNP) near the end of the Ping element, +16G/A, has become widespread amongst domesticated rice whereas the original Ping16G has been lost. The authors showed in a yeast transposition assay that the Ping16A variant transposes at a lower frequency than the original Ping16G. The authors also found that the rice strains with the highest number of mPing contain a Ping16A element inserted in a Stowaway transposable element, called Ping16A_Stow. However, the Pong element, which is known to be able to transpose mPing, is not involved in the ongoing burst as all its copies are silenced through heterochromatinization.

The investigations of Ping16A and Pong silencing are well-supported by their analysis and experiments, in the case of Ping16A. The Ping16A_Stow part is an interesting observation but there is however no mechanism which could explain how this Ping locus could be involved in the ongoing burst. The authors say in the Discussion that they did not observe any difference in the expression level of Ping at this locus (Ping16A_Stow) compared to other Ping loci. In absence of any mechanistic insight, it begs the question of whether the Ping16A_Stow locus is important for the ongoing burst or an inconsequential event which happen to be present in the rice strains with a high number of mPing elements as a consequence of a higher transposition level of the mPing/Ping/Pong TE family.

Regarding the bioinformatics: it seems ok to me but I am not really an expert in evolutionary genetics. Clearly the RelocaTE2 software is better than the read depth approach they had to use for the wild rice but since they didn't have enough sequencing depth for these strains, it should be ok as an approximation (Sup Fig 1 supports this).

Minor comments:
An explanation of introgression would be useful for people who are not expert in plant biology.

line 118: It is Figure 1e, not 1g.

Figure 3b: what do a and b above the error bars represent? Is it statistical significance?

Intro: Class II (DNA) elements: I think a classification system with only two classes is a bit silly. Why not just say DNA and retro-transposons, which are self explanatory?

"First, mPing targets genic regions but avoids exon sequences, thus minimizing harm to the host2,5." I find it difficult to imagine a mechanism that would allow a transposon to distinguish introns from exons unless it was talking to the splicing machinery. Is it possible that "avoids" is an anthropomorphism? Thus, we tend to observe intronic insertions because insertions disrupt exons. This is similar to the distribution of Hsmar1 mite in humans, which tend to be intronic (NAR, in the press).

It might be worth discussing the 16A/G polymorphism in relation to ref. 20. Depending on the concentration of transposase, a lower affinity of the protein for the transposon end can increase the rate of transposition. This phenomenon should be considered when setting up the yeast assay as the rate of transposition can be inversely proportional to the expression level.

Am I correct in thinking that Ping16A_Stow has a full length functional transposase? Does it have many AA changes compared to the canonical sequence? Has it been or will it be tested in the yeast assay?

For the reader that knows little about rice, would it be possible to include a statement in the Introduction about how similar are the 3000 strains? Something general, such as chromosome numbers, chromosomal translocations and overall sequence identity.

Reviewer #2 (Remarks to the Author):

The paper reports the identities and copy numbers of mPing, Ping and Pong variants in wild and domesticated rice genomes and relates these data to differences in the identities and activities of these elements in domesticated compared with wild rice. The paper is the first large scale survey of these elements in a wide range of rice genomes.

The authors emphasise two differences between the TE profiles of domesticated and wild rice. The first of these is the presence of the Ping16A variant in domesticated but not wild rice. They provide experimental evidence in the form of yeast transposition assays that the Ping16A variant has lower transpositional activity. The second difference is a similar profile for the Ping16A_Stow locus which again is absent in wild rice but present in some domesticates. This locus has previously been suggested to have initiated a burst of mPing activity, although the evidence for this is indirect, based only on the observation that this locus is present in rice strains with higher mPing copy numbers. On the whole the paper is therefore interesting as a description of the recent evolution of this TE family, with important implications regarding the genetic basis to changes in element activity and proliferation.

The paper is less convincing in the link made between Ping16A and Ping16A_Stow and the domestication process, the origination of these elements being described in the title as "key events ….. that occurred during rice domestication", this theme being re-emphasised in the discussion, e.g. lines 266-267. The authors do not present any evidence indicating when Ping16A and Ping16A_Stow originated, so the title of the paper is justified only if 'during domestication' is interpreted as meaning 'at any time since domestication'. The title is not justified if 'during domestication' is interpreted as that phase during the initial cultivation of rice when the genetic changes giving rise to the domestication phenotype occurred - which is the interpretation that most evolutionary biologists and all archaeologists would use.

In summary, the paper is interesting in the context of TE evolution, but it is only establishes that Ping16A and Ping16A_Stow originated in the domesticated population. It does not provide any evidence suggesting that Ping16A and Ping16A_Stow arose as a result of genetic events associated with domestication, nor that Ping16A and Ping16A_Stow influenced any genetic events involved in domestication, despite the title and discussion implying otherwise.

Reviewer #3 (Remarks to the Author):

The masnuscript from Chen et al deals with the possible association of the mPING burst and the rice

domestication. With the help of the large array of data available in the 3KRGP, the authors tried to identify the reasons of mPING burst linked to sequence change and adapatation.
While the idea is seducing and the amount of raw data available very large, the way authors analyzed them is plenty of abnormalities and the subsequent conclusions are not supported by the results. Indeed, authors compared sativa and rifupogon using different methods of analyses, and claimed that the mPing burst is related to the Ping16A mutation selection during domestication, in addition to the Ping16A_Stow potential impact. However, as the data cannot be compared, as the set of sequences is biased for the wild, and as there are no evolutionary analyses to overcome the possibility of allele sorting in the data (in addition of a lot of missing controls, see below), the conclusions drawn by the authors are erroned IMO. Thus, I would recommend a rejection of the present version of the manuscript.

Detailed comments:

- Introduction: the text is missing a lot of references in my opinion, eg line 41 to 44
- L50-52 and latter : the MITEs are associated to non-coding region of genes. However, there are no proff yet that it is a "preferential insertion" or a non-elimination through natural selection. This affiramtion of the "decision" of the TE can be feel all around the manuscript, as if the authors proposed that the TE can "decide" the way they behave... TE are only molecules, and selection acts upon them. Most of "preferential insertion" ideas has been shown latter on as more opportunistic ones (euchromatic insertion easier if no helicase eg) not eliminated by the selection (not impacting host fitness) rather than dedicated/targeted one as for Ty5 eg in yeast...
- The "strain" term is generally reserved for bacteria or yeast. For rice, please use the term "accession".
- L59-62: ok, mPing burst will probably not provoke a siRNA control/repression of Ping. But what about Ping itself ?

-Methods:
- In terms of data, authors referred to the rufipogon data as low coverage and cannot perform the same analyses as on the 3k sativa data. However, in the 3k, the coverage fluctuates from 2x to ~45x, with a mean of 14x... How do they deals with such variation in the identification of their insertion ? In addtion, the data are mapped on Nipponbare, and it has been shown in numerous papers (beginning with Schatz 2014) that the different rice subspecies may have between 5 ato 10% of genomic differences in terms of presence/absence.... FInally, authors used the filtered BAM from 3k (the re-aligned and not the pre-aligned) to detect Pong new inesrtions, but all abnormally mapped reads were already removed from those BAMs. Thus they have missed a lot of data...
- Oryza glaberrima is not a wild plant but a cultivated one. In adition, more than 4 genome assemblies are available for this plant...
- Glumipatula is glumepatula.
- L307-308: the indica group seems overrrepresented compared to others groups.
- L313-314: how Relocate deals with the low cov data ? What is its error rate ?
- L 320 and latter: the number of Ping and Pong is here overestimated
- L 334-335: how do you compute the depth variation ? Which methods did you use to confirm the statistical differences ? The CNV analyses are highly complex especially when dealing with less than 50x data and complexely mapped data such as TE...
- all along the manuscript, the tool options are not clearly defined, and reproducibility cannot be ensured. please provide all the parameters (eg L 352-353)
- A/G determination: only 2 reads to assign a genotype ???? Normally, if there are less than 5 or even 10 reads, such genotyping is irrelevant... And how do you deal with the possible A/G detected locus ? And why did you reconstruct/assemble the insertions only for rufipogon ?
- L 393: knowing the disparity of 3k data coverage, asking for a exp_cov of 50 is quite surprising...
- The term Class in TE refers to a higher group (see Wicker et al 2007).
- L 400 what is a representative sequence ?
- L405 and latter why using 2 different methods between sativa and rufipogon ? It is in this case not

possible to compare...
- L419; SpeedSeq parameters ??? And in addition, how can you compare SNP obtained using 2 different pipelines ??? Moreover, VCF file obtained by GATK UG is only a raw dataset and need a lot of refining....

-Results

-L 96: the average size is said of 500 but you assembled using velvet at 400
-L104: how did you assess the "robustness" ? Simulated data ? Data already tested by another way ?
-L 106: what is the "limited insert size" for rufipogon ?
- What is the mean value of insertion per accession ? the standard variation ?
- L 115-116: if significant, what are the p-value ?
-L 118: standard dev ?
- L 124: indeed the 3k data have less PING because the BAM used are filtered for those type of data...
-L 129: what about sequence divergence that may impair the detection and thus the results ?
- L 137-139: you said previously that you cannot identify the different insertions. How can you assess that there are no differences then ?
- L 146: how many insertions were manually scanned ?
- L 166-168: if Ping transposase is working why did not you used it as main control ? And what about different TPase as negative control ?
- L 170 and latter: you used a reference based approach, thus you are not able to identify not-reference insertions ? I mean if it is in a section of the genome not present in Nipponbare you cannot see it. Your results in addition only suggest that Ping16A_Stow may be perhaps implied in the burst, or a resulting effect of it, not the initiator...
- L 190-191: Which reference ?
- L 196: I cannot find where you check the Pong deletion derivative in methods...
- L 206-208: all your data do not show any link to domestication... only a potential strong allele sorting

Discussion: as all the results are for me not relevant or allowing any decisions, the whole discussion, especially the impact of domestication is for me to be changed.

Reviewers' comments:

Reviewer #1 (Remarks to the Author):

This is a very significant paper that addresses important questions regarding genome dynamics in rice, which is of particular interest because of its importance in the food economy of the world. Although the results are largely descriptive, they formulate further important questions, which warrant further investigation. Of course, important insights rely on posing good questions, which this work certainly does.

The authors previously published a paper (Lu, l et al, PNAS, 2017) where they characterized fours rice strains undergoing a burst of a transposable element (TE), mPing. They now analyse the genome of ~3,000 strains of domesticated and wild rice to investigate if other mechanisms could regulate the mPing burst. They analyzed the distribution of the mPing/Ping/Pong TE family in the different strains and found that a single nucleotide polymorphism (SNP) near the end of the Ping element, +16G/A, has become widespread amongst domesticated rice whereas the original Ping16G has been lost. The authors showed in a yeast transposition assay that the Ping16A variant transposes at a lower frequency than the original Ping16G. The authors also found that the rice strains with the highest number of mPing contain a Ping16A element inserted in a Stowaway transposable element, called Ping16A_Stow. However, the Pong element, which is known to be able to transpose mPing, is not involved in the ongoing burst as all its copies are silenced through heterochromatinization.

The investigations of Ping16A and Pong silencing are well-supported by their analysis and experiments, in the case of Ping16A. The Ping16A_Stow part is an interesting observation but there is however no mechanism which could explain how this Ping locus could be involved in the ongoing burst. The authors say in the Discussion that they did not observe any difference in the expression level of Ping at this locus (Ping16A_Stow) compared to other Ping loci. In absence of any mechanistic insight, it begs the question of whether the Ping16A_Stow locus is important for the ongoing burst or an inconsequential event which happen to be present in the rice strains with a high number of mPing elements as a consequence of a higher transposition level of the mPing/Ping/Pong TE family.

Regarding the bioinformatics: it seems ok to me but I am not really an expert in evolutionary genetics. Clearly the RelocaTE2 software is better than the read depth approach they had to use for the wild rice but since they didn't have enough sequencing depth for these strains, it should be ok as an approximation (Sup Fig 1 supports this).

Thank you for the supportive comments. We agree that the mechanism of how *Ping16A_Stow* may trigger *mPing* bursts is an interesting and important topic. The mechanism is currently under investigation as we stated in the discussion. We believe that it is beyond the scope of this study as discussed below.

Prior studies (Lu et al, *Proc Natl Acad Sci USA* 2017, 114: E10550–E10559) led us to hypothesize that *Ping16A_Stow* is important for the ongoing bursts and not an inconsequential event that "happens to be present in rice strains with high numbers of *mPing* elements". Data that led to this hypothesis is published and summarized below but has also been made more explicit in the text (Line 130-134). Simply stated, *Ping16A_Stow* doesn't just happen to be in accessions with high *mPing* and *Ping* copies. Rather, we determined previously that it was the only *Ping* locus in the progenitor accession prior to the start of the burst.

Lu, et al. (Lu et al, *Proc Natl Acad Sci USA* 2017, 114: E10550–E10559) demonstrated that there were 2 *mPing* bursts occurring in the accession pairs EG4/HEG4 and A123/A119 that were initiated within the past century (during very recent domestication). The results from this study under review allow us to further conclude that, among sequenced rice, bursts are only occurring in the previously described accessions (because we could not detect a burst in any of the 3,000

genomes analyzed), thus restricting *mPing* bursts to very recent domestication. This led us to hypothesize that some or all of the members of the *Ping*/*mPing* family in these accessions are responsible for the burst.

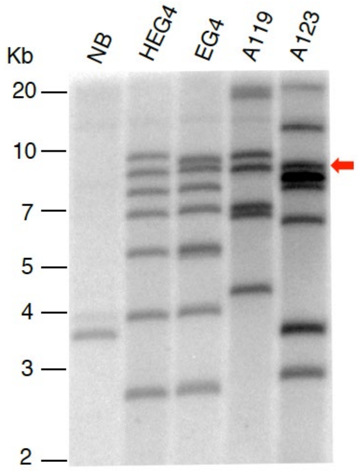

Data in the prior study (Lu et al, *Proc Natl Acad Sci USA* 2017, 114: E10550–E10559) strongly suggested that one component was *Ping16A_Stow*. Specifically, accessions A123/A119 harbor 10 and 7 *Ping* loci, respectively. Whole genome sequencing and comparative analysis allowed us to construct their last common ancestor (~100 years ago) that contained only 1 *Ping* locus –*Ping16A_Stow*. In addition, *Ping16A_Stow* is the only *Ping* locus also shared with accessions EG4/HEG4 (see Lu et al, *Proc Natl Acad Sci USA* 2017, 114: E10550–E10559, Fig 2B-where genomic DNAs were probed with the central *Ping* fragment. The red arrow denotes the presence of *Ping16A_Stow* in all four accessions. This was also demonstrated more rigorously by comparative sequence analysis). These data led us to hypothesize that *Ping16A_Stow* was involved in the burst but, clearly, more data was required.

In the study under review we present additional data to support this hypothesis, namely the presence of *Ping16A_Stow* in several other accessions with high *mPing* copy numbers. However, as clearly stated in the discussion, we do not know the mechanism of action for this important element but we can rule out the simple model that this locus encodes more transcript. Determining the actual mechanism is extremely difficult -- requiring the construction of new and/or modified rice accessions. Given that this is rice and not a unicellular organism, we strongly believe that determination of the actual mechanism is beyond the scope of this study.

Minor comments:
An explanation of introgression would be useful for people who are not expert in plant biology.

We added the definition of introgression in the Methods where we described the characterization of introgression. "Introgression was defined as an allele that is present in one subgroup but has originated from another subgroup." (Lines 532-533)

line 118: It is Figure 1e, not 1g.

This has been corrected in the revised manuscript: "All *O. rufipogon* accessions have *mPing* elements with copy numbers ranging from 1-11 (mean = 4.06, standard deviation = 2.39; Fig. 1e and Supplementary Fig. 2)." (Lines 236-238)

Figure 3b: what do a and b above the error bars represent? Is it statistical significance?

Letters ("a" and "b") above the bars indicate significant differences in transposition frequency between *mPing* variants and control. Yes, it is statistically significant with adjusted *P* value ≤ 0.05. The adjusted *P* values are based on a one-way ANOVA (*P* value = 2.37e-15, *F* value = 12.34, DF = 16) followed by a Tukey's honest significant difference (Tukey's HSD) test. The "a" category indicates no significant difference

compared to control, whereas the "b" category indicates significant difference (adjusted *P* value ≤ 0.05).

We described the statistical analysis in the figure legend and added the statistical analysis in the text that stated: "The results indicate that both the mutations adjacent to the TIRs (G16A and G17T) and 12 of 14 mutations in the TIR significantly reduced *mPing* transposition (one-way ANOVA with Tukey's HSD test, adjusted *P* value ≤ 0.05; Fig. 3b), supporting the hypothesis that this SNP (+16G/A) may have functional significance by reducing *Ping16A*'s mobility." (Lines 302-306)

Intro: Class II (DNA) elements: I think a classification system with only two classes is a bit silly. Why not just say DNA and retro-transposons, which are self explanatory?

This has been corrected in the revised manuscript to: "MITEs are nonautonomous DNA transposons that are the most common TE associated with the noncoding regions of plant genes." (Lines 93-94)

"First, mPing targets genic regions but avoids exon sequences, thus minimizing harm to the host2,5." I find it difficult to imagine a mechanism that would allow a transposon to distinguish introns from exons unless it was talking to the splicing machinery. Is it possible that "avoids" is an anthropomorphism? Thus, we tend to observe intronic insertions because insertions disrupt exons. This is similar to the distribution of Hsmar1 mite in humans, which tend to be intronic (NAR, in the press).

We cite published data demonstrating that the mechanism that significantly reduces *mPing* insertions into rice exon insertions is that *mPing* has a target preference for AT, while rice exons are much more GC rich than rice introns. The preference for *mPing* insertion into AT-rich sequences has been observed in yeast, Arabidopsis, rice, and soybean. It was also observed that *mPing*'s exon avoidance was less pronounced in soybean where the exons are less GC rich than rice (Hancock et al, *Plant Physiol* 2011, 157: 552-562). See more details in response to reviewer #3's question regarding L50-52.

We revised the manuscript to include the AT-rich insertion preference: "First, although *mPing*, like other DNA TEs, prefers genic insertions, *de novo* insertions in exons were only 14% of expected for random insertions, thus minimizing harm to the host. Exon avoidance arises from *mPing*'s extended 9-bp AT-rich insertion preference coupled with rice exon sequences that are significantly more GC rich than rice introns (51% vs. 37%)." (Lines 101-105)

It might be worth discussing the 16A/G polymorphism in relation to ref. 20. Depending on the concentration of transposase, a lower affinity of the protein for the transposon end can increase the rate of transposition. This phenomenon should be considered when setting up the yeast assay as the rate of transposition can be inversely proportional to the expression level.

Experiments in the Hancock lab show that increasing transposase expression levels in yeast results in an increase in *mPing* transposition rate (unpublished). This suggests that this element and its associated proteins do not exhibit the overexpression inhibition observed for some elements. This is consistent with weaker affinity between the transposase proteins and the TIRs resulting in less transposition. In addition, unpublished results demonstrate a linear relationship between increasing *Ping* copies and *mPing* transposition.

Am I correct in thinking that Ping16A_Stow has a full length functional transposase? Does it have many AA changes compared to the canonical sequence? Has it been or will it be tested in the yeast assay?

Yes, the transposase protein in *Ping16A_Stow* is full length and encodes an identical transposase to other *Ping* elements. Data presented in this study show that strains with only *Ping16A_Stow* have more *mPings* – a measure of the activity of the transposase. *Ping16A_Stow* also contributes to a *Ping* dosage series (in preparation) and strains with only *Ping16A_Stow* have some *mPing* transposition (in preparation)

For the reader that knows little about rice, would it be possible to include a statement in the Introduction about how similar are the 3000 strains? Something general, such as chromosome numbers, chromosomal translocations and overall sequence identity.

We added a brief summary of similarity and differences of rice accessions in the introduction: "Rice genomes are very stable: all analyzed genomes are composed of 12 chromosomes (Stein et al, *Nat Genet* 2018, 50: 285-296; Schatz et al, *Genome Biol* 2014, 15: 506), and rice subgroups share high sequence identity (> 98.9%) (Schatz et al, *Genome Biol* 2014, 15: 506). However, the genomes also exhibit extensive presence-absence variation both within (5%) and between (10%-19%) subgroups (Schatz et al, *Genome Biol* 2014, 15, 506; Wang et al, *Nature* 2018, 557: 43-49), with TEs representing more than half of this variation". (Lines 141-166).

Stein, J.C. *et al.* Genomes of 13 domesticated and wild rice relatives highlight genetic conservation, turnover and innovation across the genus Oryza. *Nat Genet* 50, 285-296 (2018).
Schatz, M.C. et al. Whole genome de novo assemblies of three divergent strains of rice, *Oryza sativa*, document novel gene space of *aus* and *indica*. *Genome Biol* 15, 506 (2014).
Wang, W. et al. Genomic variation in 3,010 diverse accessions of Asian cultivated rice. *Nature* 557, 43-49 (2018).

Reviewer #2 (Remarks to the Author):

The paper reports the identities and copy numbers of mPing, Ping and Pong variants in wild and domesticated rice genomes and relates these data to differences in the identities and activities of these elements in domesticated compared with wild rice. The paper is the first large scale survey of these elements in a wide range of rice genomes.

The authors emphasise two differences between the TE profiles of domesticated and wild rice. The first of these is the presence of the Ping16A variant in domesticated but not wild rice. They provide experimental evidence in the form of yeast transposition assays that the Ping16A variant has lower transpositional activity. The second difference is a similar profile for the Ping16A_Stow locus which again is absent in wild rice but present in some domesticates. This locus has previously been suggested to have initiated a burst of mPing activity, although the evidence for this is indirect, based only on the observation that this locus is present in rice strains with higher mPing copy numbers. On the whole the paper is therefore interesting as a description of the recent evolution of this TE family, with important implications regarding the genetic basis to changes in element activity and proliferation.

The paper is less convincing in the link made between Ping16A and Ping16A_Stow and the domestication process, the origination of these elements being described in the title as "key events ….. that occurred during rice domestication", this theme being re-emphasised in the discussion, e.g. lines 266-267. The authors do not present any evidence indicating when Ping16A and Ping16A_Stow originated, so the title of the paper is justified only if 'during domestication' is interpreted as meaning 'at any time since domestication'. The title is not justified if 'during domestication' is interpreted as that phase during the initial cultivation of rice when the genetic changes giving rise to the domestication phenotype occurred - which is the interpretation that most evolutionary biologists and all archaeologists would use.

In summary, the paper is interesting in the context of TE evolution, but it is only establishes that Ping16A and Ping16A_Stow originated in the domesticated population. It does not provide any evidence suggesting that Ping16A and Ping16A_Stow arose as a result of genetic events associated with domestication, nor that Ping16A and Ping16A_Stow influenced any genetic events involved in domestication, despite the title and discussion implying otherwise.

Thank you for the supportive comments. We agree with the reviewer and have changed the wording of the title to more accurately reflect the purview and conclusions of the study. It now reads:

"Tracking the origin of two genetic components associated with transposable element bursts during recent rice domestication"

Further, we have changed all references in the manuscript to "key events" and refer instead to "genetic components". We agree with the reviewer that an "event" is something that actually happened (during domestication) as opposed to the interpretation that *Ping16A* and *Ping16A_Stow* were rare alleles in the wild progenitor that became more abundant in lineages leading to the bursting accessions. Having said (or written!) that, this study clearly demonstrates that *mPing* bursts are restricted to the very recent events previously reported.

Reviewer #3 (Remarks to the Author):

The masnuscript from Chen et al deals with the possible association of the mPING burst and the rice domestication. With the help of the large array of data available in the 3KRGP, the authors tried to identify the reasons of mPING burst linked to sequence change and adapatation.

While the idea is seducing and the amount of raw data available very large, the way authors analyzed them is plenty of abnormalities and the subsequent conclusions are not supported by the results. Indeed, authors compared sativa and rifupogon using different methods of analyses, and claimed that the mPing burst is related to the Ping16A mutation selection during domestication, in addition to the Ping16A_Stow potential impact. However, as the data cannot be compared, as the set of sequences is biased for the wild, and as there are no evolutionary analyses to overcome the possibility of allele sorting in the data (in addition of a lot of missing controls, see below), the conclusions drawn by the authors are erroned IMO. Thus, I would recommend a rejection of the present version of the manuscript.

Thank you for the valuable comments. We revised the manuscript to address the reviewer's questions and suggestions accordingly.

Detailed comments:

- Introduction: the text is missing a lot of references in my opinion, eg line 41 to 44

New references have been added in the revised manuscript: "Eukaryotic genomes are populated with transposable elements (TEs), many attaining copy numbers of hundreds to thousands of elements by rapid amplification, called a TE burst (Feschotte et al, *Nat Rev Genet* 2002, 3: 329-41). For a TE to successfully burst it must be able to increase its copy number without killing its host or being silenced by host surveillance (Fultz et al, *Curr Opin Plant Biol* 2015, 27: 67-76; Feschotte et al, *Annu Rev Genet* 2007, 41: 331-68). However, because the vast majority of TE bursts have been inferred after the fact – via computational analysis of whole genome sequence – the stealth features they require for success have remained largely undiscovered (Fultz et al, *Curr Opin Plant Biol* 2015, 27: 67-76; Huang et al, *Annu Rev Genet* 2012, 46: 651-75)." (Lines 84-90)

Feschotte, C., Jiang, N. & Wessler, S.R. Plant transposable elements: where genetics meets genomics. Nat Rev Genet 3, 329-41 (2002).
Fultz, D., Choudury, S.G. & Slotkin, R.K. Silencing of active transposable elements in plants. Curr Opin Plant Biol 27, 67-76 (2015).
Feschotte, C. & Pritham, E.J. DNA transposons and the evolution of eukaryotic genomes. Annu Rev Genet 41, 331-68 (2007).
Huang, C.R., Burns, K.H. & Boeke, J.D. Active transposition in genomes. Annu Rev Genet 46, 651-75 (2012).

- L50-52 and latter : the MITEs are associated to non-coding region of genes. However, there are no proff yet that it is a "preferential insertion" or a non-elimination through natural selection. This affiramtion of the "decision" of the TE can be feel all around the manuscript, as if the authors proposed that the TE can "decide" the way they behave... TE are only molecules, and selection acts upon them. Most of "preferential insertion" ideas has been shown latter on as more opportunistic ones (euchromatic insertion easier if no helicase eg) not eliminated by the selection (not impacting host fitness) rather than dedicated/targeted one as for Ty5 eg in yeast...

Contrary to the reviewer's comments, the mechanism of *Ping/mPing* target preference in noncoding regions is known and was documented in prior studies, which were referenced in the Introduction (Lines 101-105). Given that this point confused two reviewers, we expanded the introduction to include a brief summary of the mechanism. However, when limited by word count, we have to rely on the readers (and one would think the reviewers) to have read at least the abstracts of cited papers. Simply stated, it is due to the *Ping/mPing* target sequence preference (AT rich, whereas rice exons are GC rich) and does not reflect selection against exon insertions.

For the reviewers, we have presented details from the cited papers:
-*Ping/mPing* has an extended 9-bp AT rich insertion preference (see Naito et al, 2006, *Proc Natl Acad Sci USA* 103: 17620-25; Naito et al, *Nature* 2009 461: 1130-34). Rice exons are significantly more GC rich than rice introns [51% vs 37%, see Yu et al (2002) *Science* 296: 79–92].

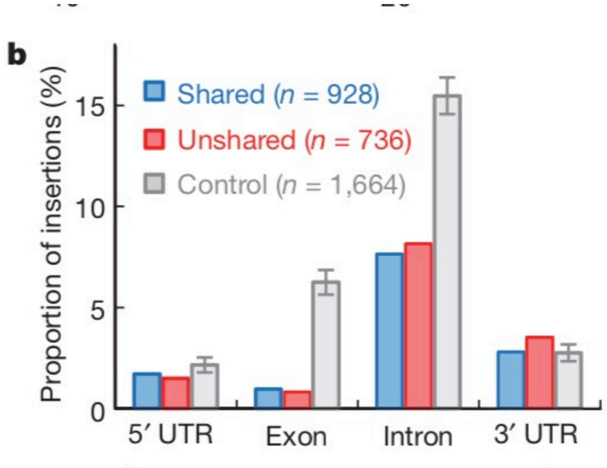

A preference for de novo intron over exon insertions was demonstrated in two ways – both published. First, analysis of ~1600 de novo *mPing* insertion sites (virtually all somatic, heterozygous, ruling out selection because they just happened) revealed only 14% of the expected number for random exon insertions while insertions into introns is much higher - 51% of expected (see Naito et al, *Nature* 2009 461: 1130-34, Fig1b is shown).

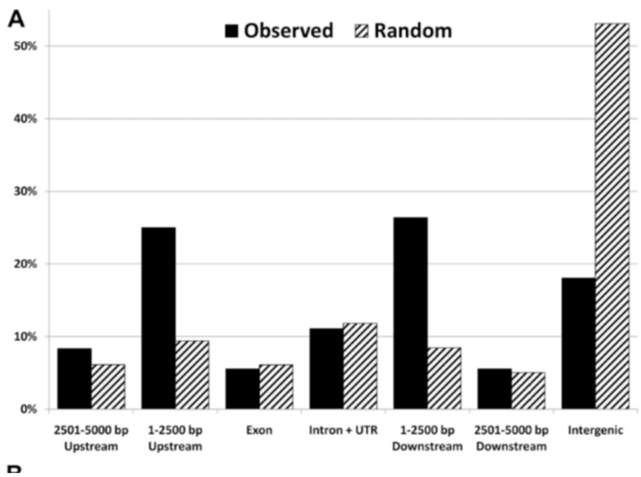

Second, when the *Ping*/*mPing* elements were analyzed in transgenic soybean – (where exons are 43% GC), we observed a significantly higher frequency of exon insertions than in rice (see Hancock et al, *Plant Physiol* 2011, 157: 552-562, see figure 8b and details in the discussion of this paper). So, to reviewer #3 -yes there is published proof – we are data-driven scientists and resent being accused of believing in magic. There is no magic mechanism or "feeling" that *mPing* has - of recognizing exons in DNA, rather, just straight forward (boring!) biochemical sequence preference coupled with host genome features. We suspect that the *mPing* burst in rice reflects a fortuitous association between the sequence preference of the *Ping* transposase and the sequence features of the rice genome. Stated another way, species without GC rich exons (like soybean and most other higher plants) would not survive an *mPing* burst. The burst can be sustained in rice because it is less mutagenic (again, unlike in soybean).

We revised the manuscript to include the AT-rich insertion preference: "First, although *mPing*, like other DNA TEs, prefers genic insertions, *de novo* insertions in exons were only 14% of expected for random insertions, thus minimizing harm to the host. Exon avoidance arises from *mPing*'s extended 9-bp AT-rich insertion preference coupled with rice exon sequences that are significantly more GC rich than rice introns (51% vs. 37%)." (Lines 101-105)

Naito, K. et al. Dramatic amplification of a rice transposable element during recent domestication. *Proc Natl Acad Sci USA* 103, 17620-5 (2006).
Naito, K. et al. Unexpected consequences of a sudden and massive transposon amplification on rice gene expression. *Nature* 461, 1130-4 (2009).
Yu, J. et al. A draft sequence of the rice genome (*Oryza sativa* L. ssp. *indica*). *Science* 296, 79-92 (2002).
Hancock, C.N. et al. The rice miniature inverted repeat transposable element *mPing* is an effective insertional mutagen in soybean. *Plant Physiol* 157, 552-62 (2011).

- The "strain" term is generally reserved for bacteria or yeast. For rice, please use the term "accession".

This has been corrected in the revised manuscript by using the term "accession" throughout.

- L59-62: ok, mPing burst will probably not provoke a siRNA control/repression of Ping. But what about Ping itself ?

To date, we have not identified a situation where the *Ping* insertion site has triggered siRNAs to *Ping* genic regions (Lu et al, *Proc Natl Acad Sci USA* 2017, 114: E10550–E10559). It is likely that as *Ping* copy numbers increase, a *Ping* element will insert into a region that will generate antisense RNAs and lead to the production of siRNAs that silence *Ping* expression. To date, this has not been detected.

-Methods:
- In terms of data, authors referred to the rufipogon data as low coverage and cannot perform

the same analyses as on the 3k sativa data. However, in the 3k, the coverage fluctuates from 2x to ~45x, with a mean of 14x... How do they deals with such variation in the identification of their insertion ? In addtion, the data are mapped on Nipponbare, and it has been shown in numerous papers (beginning with Schatz 2014) that the different rice subspecies may have between 5 ato 10% of genomic differences in terms of presence/absence.... FInally, authors used the filtered BAM from 3k (the re-aligned and not the pre-aligned) to detect Pong new inesrtions, but all abnormally mapped reads were already removed from those BAMs. Thus they have missed a lot of data...

The sequencing depth of 3,000 rice accessions ranges from 4.03 to 63.93x coverage with an average depth of coverage of 14x. One accession, IRIS_313_8921, was an exception with very low depth of 0.02. This accession was treated as missing data in this study. The sequencing depth of *O. rufipogon* accessions was on average 11X, ranging from 3.78 to 36.97X. The sequencing depth of both rice and *O. rufipogon* accessions are sufficient for detecting TE insertions and transpositions by RelocaTE2 because the tool can recover over 80%-90% of TE insertions using paired-end reads of 3-fold depth of coverage (Chen et al, *PeerJ* 2017, 5: e2942).

The short insert size (~300 bp) of *O. rufipogon* libraries is the primary limitation that prevents distinguishing *Ping* and *Pong* from *mPing*. However, the situation is even worse where there is low depth of coverage because fewer paired-end reads are available to distinguish *Ping* and *Pong* from *mPing*. We stated in the text "Insertion sites and copy numbers for *mPing*, *Ping* and *Pong* were also identified for 48 *O. rufipogon* accessions, but only the read-depth method was used because of the limited insert size of the libraries" (Lines 206-208). In the Methods, we stated "the short insert size and insufficient read depth of *O. rufipogon* sequencing libraries prevented distinguishing *Ping* and *Pong* insertions from *mPing*." (Lines 550-551)

We agree that the 5-10% of genomic differences among rice subspecies may have impact on the characterization of *mPing*/*Ping*/*Pong* insertions. However, comparison between RelocaTE2 and the read-depth method (Supplementary Figure 1) reveals that the impact is very limited. For the major findings (two genetic components *Ping16A* and *Ping16A_Stow*) genotyping approaches were used to obtain the genotype of these *Ping* variants in rice and wild rice accessions. The genotyping approaches are not sensitive to these genomic differences.

The reviewer may have misunderstood our methods to characterize *mPing*, *Ping* and *Pong* insertions. Raw reads were used to characterize *mPing*, *Ping*, and *Pong* using RelocaTE2. BAM files were used in cases where a known locus was genotyped (such as the *Stowaway* element on chromosome 1). To clarify, the Methods have been revised as follows: "The *mPing*, *Ping*, and *Pong* insertion sites across the 3,000 rice genomes were characterized with RelocaTE2 (aligner = BLAT mismatch = 1 len_cut_trim = 10) using the raw reads for each accession obtained from NCBI SRA." (Lines 536-538)

Chen, J., Wrightsman, T.R., Wessler, S.R. & Stajich, J.E. RelocaTE2: a high resolution transposable element insertion site mapping tool for population resequencing. *PeerJ* 5, e2942 (2017).

- Oryza glaberrima is not a wild plant but a cultivated one. In adition, more than 4 genome assemblies are available for this plant...

This has been corrected in the revised manuscript.

"The raw reads of *Oryza glaberrima*, *Oryza glumaepatula* (also known as *Oryza glumipatula*), and *Oryza meridionalis* were obtained from NCBI SRA projects accession numbers SRR1712585, SRR1712910, and SRR1712972." (Lines 479-481)

"*O. glaberrima*, *Oryza glumaepatula*, and *O. meridionalis* were treated as outgroups." (Line 782)

- Glumipatula is glumepatula.

The taxonomic nomenclature for *Oryza glumipatula* is still in revision (e.g. Vaughan et al, *Curr. Opin. Plant Biol* 2003, 6: 139-46). NCBI Taxonomy uses *Oryza glumipatula* as the scientific name while an alternative name, *Oryza glumaepatula,* is widely used in the literature. To clarify, we revised the manuscript to use *Oryza glumaepatula* as the primary name and include *Oryza glumipatula* as its alternative name: "The raw reads of *Oryza glaberrima*, *Oryza glumaepatula* (also known as *Oryza glumipatula*), and *Oryza meridionalis* were obtained from NCBI SRA projects accession numbers SRR1712585, SRR1712910, and SRR1712972." (Lines 479-481)

Vaughan, D.A., Morishima, H. & Kadowaki, K. Diversity in the *Oryza* genus. *Curr Opin Plant Biol* 6, 139-46 (2003).

- L307-308: the indica group seems overrrepresented compared to others groups.

This skewed sampling is because nearly half (1,651/3,000) of sequenced and analyzed rice accessions are *indica*. We used only those accessions having major ancestral components over 99.99% to perform local ancestry analysis. The numbers of individuals in each subgroup of this filtered dataset reflect their proportion in 3,000 samples.

We tested whether the numbers of individuals in the reference panel may have impacted our results. By using 30 individuals in each subgroup, similar results were obtained. The only difference is that 32 instead of 30 admixed accessions have *japonica* backgrounds at *Ping16A* loci. The numbers in Table 1 have been retained because they represent a conservative estimate of introgression.

- L313-314: how Relocate deals with the low cov data ? What is its error rate ?

We reported this more comprehensively in the RelocaTE2 paper (Chen et al, *PeerJ* 2017, 5: e2942). Briefly, RelocaTE2 is able to recover over 80-90% of TE insertions using low coverage data (as low as 3-fold genome coverage). Therefore, we analyzed rice accessions with 3-fold or more coverage. This includes 2,999 rice accessions. The only exception is IRIS_313_8921 which has 0.02-fold genome coverage and this has been excluded from further analysis. The error rate (false positive) of RelocaTE2, based on simulation analysis, is less than 1% even when using low coverage data (Chen et al, *PeerJ* 2017, 5: e2942).

Chen, J., Wrightsman, T.R., Wessler, S.R. & Stajich, J.E. RelocaTE2: a high resolution transposable element insertion site mapping tool for population resequencing. *PeerJ* 5, e2942 (2017).

- L 320 and latter: the number of Ping and Pong is here overestimated

Due to the complexity of repeat content in the genome we agree that the RelocaTE2 method may underestimate or overestimate *Ping* and *Pong* copy numbers in some accessions. We compared the copy numbers estimated using RelocaTE2 and a read-depth method (as shown in Supplementary Figure 1). The results between these two methods are well correlated. Because we thought that such variations could impact the conclusion of this study we used genotyping approaches to determine the presence and absence of these *Ping* variants (*Ping16A* and *Ping16A_Stow*) in rice and *O. rufipogon* accessions. Therefore, the variation of copy numbers estimated using RelocaTE2 or the read-depth method does not impair our conclusions.

- L 334-335: how do you compute the depth variation ? Which methods did you use to confirm the statistical differences ? The CNV analyses are highly complex especially when dealing with less than 50x data and complexely mapped data such as TE...

In the initial submission, we computed an overall read depth for *mPing/Ping/Pong* and compared it to the genome-wide read depth to obtain an approximate estimation of *mPing/Ping/Pong* copy number for each accession. In the revised manuscript, we refined our method to consider the depth variation in *mPing/Ping/Pong* elements. We computed the read depth of each 50-bp window for *mPing/Ping/Pong*. The copy number of each 50-bp window was defined as the ratio of depth of each 50-bp window to genome-wide read depth. Approximate estimation of *mPing/Ping/Pong* copy numbers were estimated as an average copy number of all 50-bp windows. To confirm the statistical differences, we performed a one-sample *t*-test to determine whether the mean read depth of 50-bp windows in a given element was equal to genome-wide read depth. We compared the copy numbers estimated with two methods. The results are well correlated (see the figures below). We revised the manuscript to include the copy numbers estimated with the refined method (Lines 202-204, 209-211, 236-238 in results, Lines 552-620 in the Methods, Supplementary Figure 2, and Supplementary Table 2).

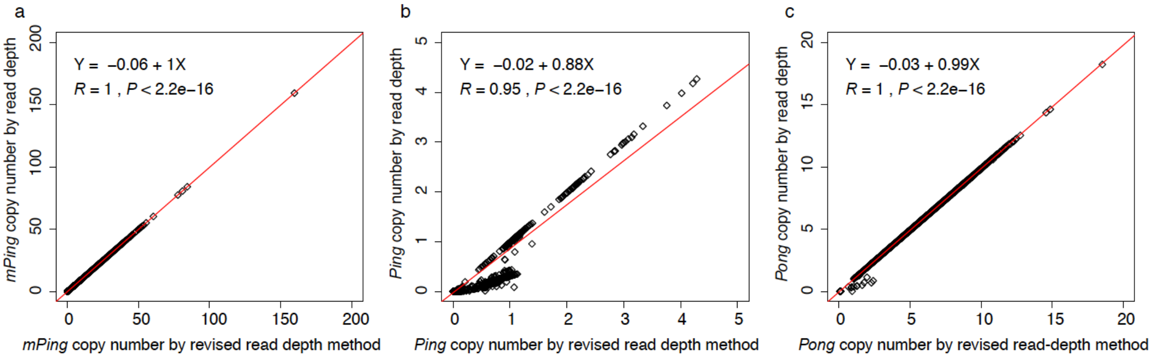

- all along the manuscript, the tool options are not clearly defined, and reproducibility cannot be ensured. please provide all the parameters (eg L 352-353)

We apologize for the inconvenience. In cases where no parameters were described, the default parameters were used. We have updated the text to specify exactly what these defaults are and what parameters were used. To enhance the reproducibility of this study all scripts have been deposited into github repository which is referenced in the manuscript [https://github.com/stajichlab/Dynamic_rice_publications].

- A/G determination: only 2 reads to assign a genotype ???? Normally, if there are less than 5 or even 10 reads, such genotyping is irrelevant... And how do you deal with the possible A/G detected locus ? And why did you reconstruct/assemble the insertions only for rufipogon ?

1, *only 2 reads to assign a genotype ???? Normally, if there are less than 5 or even 10 reads, such genotyping is irrelevant*
The genotyping method we used is quite different than a genome-wide SNP genotyping method as explained below. We tested 2 reads and a more stringent parameter, 5 reads as cutoffs. Both parameters resulted in the same conclusion that *Ping16A* is not present in *O. rufipogon* but is widely distributed in *japonica* rice. We decided to use 2 reads because it allowed retaining more data points in the analyses.

For the 3k rice accessions, we used a locus-specific method that analyzes *Ping*-containing reads from a particular locus to genotype the +16G/A in *Ping*. The

method is based on two observations: 1, all *Ping*-containing reads used were from a known locus because the reads covered both the *Ping* element and the flanking sequence; 2, the genotype of +16G/A at all *Ping* loci in rice accessions tested were homozygous. Therefore, even though there are only 2 reads supporting a genotype, the chance that both reads have sequencing errors at the +16G/A position is very small. We analyzed the genotype of +16G/A in rice using 5 reads instead of 2 as a cutoff. The results are consistent for these two parameters (detailed numbers are listed in the tables below). The only difference is that some information is lost when using a more stringent parameter (5 reads).

For *O. rufipogon* accessions, because we were unable to distinguish *Ping* from *mPing* loci, the locus-specific method used for rice was not applicable. Instead, pooled reads from *mPing*, *Ping*, and *Pong* were used to genotype +16G/A of the *Ping* element. All but one *O. rufipogon* accessions(W1230) have "G" at +16G/A. In addition, we were able to trace the locations of "A"-supporting reads to an *mPing* locus. Therefore, these findings led us to conclude that *Ping16A* is not present in *O. rufipogon*. These details are described in the Methods (Lines 697-704).

2, *how do you deal with the possible A/G detected locus*
All genotyped *Ping* loci are homozygous.

3, *why did you reconstruct/assemble the insertions only for rufipogon ?*
We assembled an *mPing* locus in the *O. rufipogon* accession W1230 to confirm that all "A" supporting reads were from the *mPing* locus, not from *Ping*.

Table *Ping* variants genotyped using 2 reads as a cutoff

Subgroups	Number of strains	Number of strains with *Ping*	*Ping* variants	
			Number of strains with *Ping16G*	Number of strains with *Ping16A*
O. sativa	3,000	199 (6.6%)	31	154
-*indica*	1,651	20 (1.2%)	8	9
-*aus/boro*	189	28 (14.8%)	19	0
-temperate *japonica*	250	61 (24.4%)	1	61
-tropical *japonica*	335	51 (15.2%)	0	51
-*aromatic*	65	0 (0%)	0	0
-admixed	510	39 (7.6%)	3	33
O. rufipogon	48	21 (43.7%)	21	0
-*Or-I*	13	7 (53.8%)	7	0
-*Or-II*	23	10 (43.4%)	10	0
-*Or-IIIa*	6	2 (33.3%)	2	0
-*Or-IIIb*	6	2 (33.3%)	2	0

Table *Ping* variants genotyped using 5 reads as a cutoff

Subgroups	Number of strains	Number of strains with *Ping*	*Ping* variants	
			Number of strains with *Ping16G*	Number of strains with *Ping16A*
O. sativa	3,000	199 (6.6%)	11	137
-*indica*	1,651	20 (1.2%)	4	7

-*aus/boro*	189	28 (14.8%)	6	0
-temperate *japonica*	250	61 (24.4%)	1	59
-tropical *japonica*	335	51 (15.2%)	0	42
-*aromatic*	65	0 (0%)	0	0
-admixed	510	39 (7.6%)	0	29
O. rufipogon	48	21 (43.7%)	21	0
-*Or-I*	13	7 (53.8%)	7	0
-*Or-II*	23	10 (43.4%)	10	0
-*Or-IIIa*	6	2 (33.3%)	2	0
-*Or-IIIb*	6	2 (33.3%)	2	0

- L 393: knowing the disparity of 3k data coverage, asking for a exp_cov of 50 is quite surprising...

We appreciate this concern and addressed this by correcting the revised manuscript. Note that expected coverage is just a parameter for the local assembly approach but we reanalyzed the data using "-exp_cov auto" instead of "-exp_cov 50" to let velvet estimate the expected coverage. We also used "-ins_length 500" instead of "-ins_length 400" to match the average insert size of 500 bp in the dataset. We assembled similar numbers of full-length *mPing* elements and confirmed the presence of four *mPing* subtypes in 3,000 rice accessions (see the table below).

Velvetg parameters	"-ins_length 400" & "-exp_cov 50"	"-ins_length 500" & "-exp_cov auto"
Assembled Full-length *mPing*	11136	11595
mPingA	5324	5652
mPingB	4639	4736
mPingC	938	957
mPingD	234	249
Unknown subtype*	1	1

* This unknown subtype may represent a new deletion derivative of *Ping* or *mPing*, however has not been amplified. The sequence covering the breakpoint of these four *mPing* subtypes were not present in the unknown subtype.

- The term Class in TE refers to a higher group (see Wicker et al 2007).

"Class" has been replaced by "subtype" in the revised manuscript.

- L 400 what is a representative sequence ?

In each subgraph, the *mPing* sequences are similar to each other and show only substitutions or small indels. Therefore, we extracted one sequence as a representative sequence from each subgraph to align with four known subtypes of *mPing*.

- L405 and latter why using 2 different methods between sativa and rufipogon ? It is in this case not possible to compare...

Due to the short insert size of *O. rufipogon* reads it is impossible to assemble full-length *mPings* from many accessions. Therefore we used a reference-based method

to genotype the four subtypes in *O. rufipogon* and confirmed their presence. We did not compare the frequency of the four *mPing* subtypes or other related quantitative data between rice and *O. rufipogon.* We believe that this qualitative examination to count the number of *mPing* subtypes is valid when applying different methods to the separate populations.

- L419; SpeedSeq parameters ??? And in addition, how can you compare SNP obtained using 2 different pipelines ??? Moreover, VCF file obtained by GATK UG is only a raw dataset and need a lot of refining....

SpeedSeq parameters have been added in the revised manuscript: "Sequencing reads were aligned to OsChr3 and OsChr4 with SpeedSeq v 0.1.042 (align -t 24 -R "@RG\tID:id\tSM:sample\tLB:library"), which uses BWA (mem -k 19 -w 100 -T 30) to align reads, Sambamba43 (-M 20) to sort alignments, and SAMBLASTER44 (-c 2 -m 20) to mark PCR duplicates." (Lines 604-608)

For 3k data, we obtained the SNP data from 3KRGR and genotyped four temperate *japonica* accessions with a subset of these SNPs to construct the phylogenetic tree of temperate *japonica* (shown in Figure 2c). For *O. rufipogon*, we generated SNP data from raw reads to construct the phylogenetic tree of *O. rufipogon* accessions (shown in Supplementary Figure 2). SNP data obtained using these 2 different pipelines was not compared.

Detailed methods and parameters for refining SNPs have been added in the revised manuscript. Additional filters were applied to SNPs after GATK UG though the Methods did not spell out the parameters applied. These methods have been explained in the revision in more detail: "Filtering parameters (QD < 2.0, MQ < 40.0, FS > 60.0, AF < 0.05, HaplotypeScore > 13.0, MQRankSum < -12.5, ReadPosRankSum < -8.0, MQ0 >= 4 && ((MQ0/(1.0*DP)) > 0.1), QUAL < 30.0, DP < 6, DP > 5000, HRun > 5) were used to retain high quality SNPs using GATK VariantFiltration. Only homozygous SNPs that did not overlap with repetitive sequences were used in the phylogenetic analysis." (Lines 755-778)

-Results

-L 96: the average size is said of 500 but you assembled using velvet at 400

This has been addressed as described in the response to the question regarding L 393. We used "-ins_length 500" instead of "-ins_length 400" to match the average insert size of 500 bp in the dataset.

-L104: how did you assess the "robustness" ? Simulated data ? Data already tested by another way ?

We tested both methods on simulated datasets. The testing results of RelocaTE2 have been reported in Chen et al, *PeerJ* 2017, 5: e2942. The testing results of the read-depth method have been added to the revised manuscript as Supplementary Figure 7. These data suggest that both methods are robust based on testing simulated datasets. For the 3k datasets, the copy numbers of *mPing*, *Ping*, and *Pong* estimated using these two methods were well correlated. Therefore, we believe that it is accurate to state that these methods are robust estimators of *mPing*, *Ping*, and *Pong* copy numbers.

We revised the text to clarify the statement: "Outputs from the RelocaTE2 and read-depth methods were well correlated (Pearson's correlation, *R* = 0.97, *P* < 2.2e-16 for *mPing*; *R* = 0.82, *P* < 2.2e-16 for *Ping*; *R* = 0.66, *P* < 2.2e-16 for *Pong*; Supplementary Fig. 1) indicating that both methods to estimate approximate *mPing*, *Ping*, and *Pong* copy numbers in the 3,000 rice accessions were robust." (Lines 202-206)

Chen, J., Wrightsman, T.R., Wessler, S.R. & Stajich, J.E. RelocaTE2: a high resolution transposable element insertion site mapping tool for population resequencing. *PeerJ* 5, e2942 (2017).

-L 106: what is the "limited insert size" for rufipogon ?

The insert size of *O.* rufipogon sequences used in this study varies from 89 to 454 bp with an average of ~300 bp. The short insert size prevented us from distinguishing *Ping* and *Pong* insertions from *mPing*.

- *What is the mean value of insertion per accession ? the standard variation ?*

The mean and standard deviation (std) of *mPing*/*Ping*/*Pong* in each accession has been added to Supplementary Table 2. We revised the manuscript to cite Supplementary Table 2: "In total, 195 *mPing*s, 25 *Ping*s, and 125 *Pong*s were estimated to be present in the 48 *O. rufipogon* accessions (Supplementary Table 2, Fig. 1e-g, and Supplementary Fig. 2)." (Lines 209-211)

- L 115-116: if significant, what are the p-value ?

The statistical test and *P* value were initially described in Supplementary Figure 3. We added the statistical test and *P* value in the text and stated the result : "Temperate *japonica* accessions do, however, have significantly more *mPing* elements (~30.5/accession) than tropical *japonica* (~2.6/accession), *indica* (~8.2/accession), or *aus*/*boro* (~3.8/accession) (one-way ANOVA with Tukey's HSD test, adjusted *P* value < 2e-16; Supplementary Table 3 and Supplementary Fig. 3).". (Lines 217-234)

-L 118: standard dev ?

The mean and standard deviation of *mPing* in *O. rufipogon* has been added in the revised manuscript: "All *O. rufipogon* accessions have *mPing* elements with copy numbers ranging from 1-11 (mean = 4.06, standard deviation = 2.39; Fig. 1e and Supplementary Fig. 2)." (Lines 234-236)

- L 124: indeed the 3k data have less PING because the BAM used are filtered for those type of data...

There appears to be a misunderstanding in the Methods. Our approach with RelocaTE2 starts with raw sequence reads (FASTQ) obtained for every accession from NCBI SRA. We are not limited to the BAM files provided by the 3kRGP. The steps to identify *Ping* insertions use RelocaTE2 with raw sequence reads, not precomputed BAM. Second, *Ping* copy numbers in each rice accession were estimated using a read-depth method that evaluates the number of sequence reads found to contain partial *Ping* sequences compared to the genome-wide depth of coverage average to get a normalized ratio that reflects the approximate *Ping* copy number. Although the read-depth method and RelocaTE2 do not agree every time, they are very consistent throughout the examined 3,000 samples (*R* = 0.82, *P* < 2.2e-16). The Methods have been revised to clarify the statement: "The *mPing*, *Ping*, and *Pong* insertion sites across the 3,000 rice genomes were characterized with RelocaTE2 (aligner = BLAT mismatch = 1 len_cut_trim = 10) using raw reads that were obtained from NCBI SRA." (Lines 536-538)

-L 129: what about sequence divergence that may impair the detection and thus the results ?

We agree that sequence divergence is a factor that may impact the detection of TE insertions. This is one of most important problems solved with RelocaTE2. Mismatches were allowed when aligning reads to *mPing*/*Ping*/*Pong* (1 mismatch) or

their flanking sequences (1 or more mismatches as long as the reads are uniquely mapped) in RelocaTE2. This enables detection of TE insertions in rice or wild rice accessions with very diverse backgrounds (Fig. 3B in Chen et al, *PeerJ* 2017, 5: e2942). This is also supported by the positive correlation between the estimated copy numbers of RelocaTE2 and the read-depth method (Supplementary Figure 1).

Chen, J., Wrightsman, T.R., Wessler, S.R. & Stajich, J.E. RelocaTE2: a high resolution transposable element insertion site mapping tool for population resequencing. *PeerJ* 5, e2942 (2017).

- L 137-139: you said previously that you cannot identify the different insertions. How can you assess that there are no differences then ?

We cannot distinguish *Ping* from *mPing* in *O. rufipogon*. Therefore, we used all reads from *mPing* and *Ping* to genotype +16G/A in *O. rufipogon*. We found that all the *mPing*/*Ping* reads in *O. rufipogon* have "G" at +16G/A. While we cannot distinguish if these are the autonomous (*Ping*) element or the MITE (*mPing*) there is no evidence (except the one instance discussed below) for the 16A in the *O. rufipogon* populations.

Only accession W1230 has evidence for a '16A'. We analyzed the locations of these A-supporting reads in the genome. The results indicated that all were from a single *mPing* or *Ping* locus. We then assembled the reads that were associated with the locus and determined that all the A-supporting reads were from an *mPing* not a *Ping* locus. This is described in the Methods: "Accessions that have two or more reads supporting both G and A were further analyzed to clarify whether the *Ping16A* is present in these genomes. For example, accession W1230 had both G (288 reads) and A (23 reads) at the +16G/A SNP. These A-supporting reads and their mates were extracted from W1230 sequences and aligned to pseudogenomes that have W1230 *mPing* or *Ping* inserted in MSU7. All of these A-supporting reads were uniquely aligned to *mPing* locus Chr3:25526483-25526485 that contains a 430 bp *mPingC* element successfully assembled from locus-specific paired-end reads, suggesting these A-supporting reads were from *mPing* not from *Ping*." (Lines 697-704)

- L 146: how many insertions were manually scanned ?

We performed local ancestry assignment to identify introgressions in these accessions of interest with RFMix v2.03. Regions of interest were manually scanned in the results of RFMix. This approach is described in the Method: "Local ancestry assignment was performed on accessions of interest with RFMix v2.03 using default parameters. Regions of interest were manually inspected in the results of RFMix." (Lines 519-532)

- L 166-168: if Ping transposase is working why did not you used it as main control ? And what about different TPase as negative control ?

Good question! Unfortunately, the *Ping* TPase catalyzes much lower transposition rates in yeast requiring a very difficult version of the assay with large plates and longer incubation times (Hancock et al, *Mob DNA* 2010, 1: 5). This was incorrectly stated in the initial submission as "Although *Pong* transposase was used in this experiment to facilitate the yeast transposition assays, its catalytic activity is almost indistinguishable from *Ping* transposase". We revised the statement: " Although *Pong* transposase, which was shown previously to catalyze higher transposition frequency than *Ping*, was used in this experiment to facilitate the yeast transposition assays, its catalytic mechanism is likely indistinguishable from *Ping* transposase" (Lines 305-308). The difference in activity between the *Ping* and *Pong* proteins was shown to be due to the presence of a stronger NLS in the *Pong* ORF1 protein (Payero

et al, *Journal of the South Carolina Academy of Science* 2016, 14: 1-6). Thus, we did the bulk of the experiments with a hybrid version of ORF1 that contains the *Ping* ORF1 DNA binding domain (N-terminal region), a strong NLS, and the protein interaction domain (C-terminal region). To confirm that the key results for the +16G/A SNP were not an artifact, we tested this directly using *Ping* transposase and provided it as Supplemental Figure 5.

We are not sure what the reviewer means by using a different TPase as a control. There is no evidence that transposases from other unrelated elements are cable of mobilizing *mPing*. Previous publications have shown that mutated versions of the ORF1 and TPase proteins have no activity (Hancock et al, *Mob DNA* 2010, 1: 5).

Hancock, C.N., Zhang, F. & Wessler, S.R. Transposition of the *Tourist*-MITE *mPing* in yeast: an assay that retains key features of catalysis by the class 2 *PIF/Harbinger* superfamily. *Mob DNA* 1, 5 (2010).
Payero, L., Outten, G., Burckhalter, C.E. & Hancock, C.N. Alteration of the *Ping* and *Pong* ORF1 proteins allows for hyperactive transposition of *mPing*. *Journal of the South Carolina Academy of Science* 14, 1-6 (2016).

- L 170 and latter: you used a reference based approach, thus you are not able to identify not-reference insertions ? I mean if it is in a section of the genome not present in Nipponbare you cannot see it. Your results in addition only suggest that Ping16A_Stow may be perhaps implied in the burst, or a resulting effect of it, not the initiator...

We agree that we might miss some insertions due to the absence of their insertion sites in the reference genome. This is a challenging issue for almost all studies (not just ours) that identify TE insertions and structural variations in genomes. We validated our methods by comparing the copy number results between RelocaTE2 and the read-depth method. For *Ping16A_Stow*, we genotyped the *Ping16A_Stow* locus to determine whether a rice or wild rice accession has a wild type allele, the *Stowaway* element alone, or *Ping* inserted into the *Stowaway* element (also known as *Ping16A_Stow*).

Although *Ping16A_Stow* is found in almost all high-copy-number rice accessions, it is also present in accessions with fewer *mPings*. In the previous study (Lu et al, *Proc Natl Acad Sci USA* 2017, 114: E10550–E10559), we showed that *Ping16A_Stow* is the only shared *Ping* locus among four rice accessions undergoing very recent bursts. Further, and more importantly, *Ping16A_Stow* is the only *Ping* locus present in the last common ancestor of A123 and A119 in addition to 23 *mPings*. Therefore, we concluded that *Ping16A_Stow* was present at the start of the burst, not as a result of the burst. These results along with the correlation of *Ping16A_Stow* with higher *mPing* copy numbers in 3,000 rice accessions strongly suggest that *Ping16_Stow* plays an important role in initiating the *mPing* bursts (see more details in response to reviewer #1's comments).

Lu, L. et al. Tracking the genome-wide outcomes of a transposable element burst over decades of amplification. *Proc Natl Acad Sci USA* 114, E10550–E10559 (2017).

- L 190-191: Which reference ?

Our previous study (Lu et al, *Proc Natl Acad Sci USA* 2017, 114: E10550–E10559) demonstrated that *Pong* is silenced in Nipponbare as well as in four temperate *japonica* rice undergoing bursts. Data from the current study extend these findings and suggests that *Pong* was silenced in *O. rufipogon* and has been stably silenced in domesticated rice. We revised the sentence in the text to include the reference " Data from this study extend previous findings (Lu et al, *Proc Natl Acad Sci USA* 2017, 114: E10550–E10559) and suggest that *Pong* was silenced in *O. rufipogon* and has been stably silenced in domesticated rice." (Lines 342-343)

Lu, L. et al. Tracking the genome-wide outcomes of a transposable element burst over decades of amplification. *Proc Natl Acad Sci USA* 114, E10550–E10559 (2017).

- L 196: I cannot find where you check the Pong deletion derivative in methods...

Thank you for pointing this out. We have added details to the Methods about element detection: "The presence and absence of *mPing*, *Ping*, and *Pong* were also confirmed with manual inspection. Briefly, *mPing*, *Ping*, and *Pong*-associated reads were extracted and aligned to the elements as described above. The sequence coverage of *mPing*, *Ping*, and *Pong* were inspected using heatmap and Integrative Genomics Viewer (IGV) v2.3.0. Only accessions showing sequence coverage across a given element (generally needs ≥ 70% of elements covered) were defined as accessions having this element. This approach was also used to identify four *Aus/boro* accessions that have a *Ping* locus (Chr11:25822230-25802232) that was not identified with RelocaTE2." (Lines 621-630)

- L 206-208: all your data do not show any link to domestication... only a potential strong allele sorting

While we do not know exactly how these six rice accessions accumulated more *Pong* elements in their genomes, the data presented strongly suggests that it was not due to transposition. This conclusion is significant because it supports the contention that *Pong* has been stably silenced in all accessions analyzed. This is also supported by the fact that the six accessions with high *Pong* copy number (1) have few *mPings* (active *Pong* can catalyze *mPing* transposition/increase in copy number in cell culture and transgenic plants) and (2) do not show increased copies of other silenced rice TEs (as would be expected if these strains lost epigenetic regulation at some point in their lineage(s)). Allele sorting is a possibility for more *Pongs* in these accessions. Again, the point to be emphasized is that *Pong* is silenced in *O. rufipogon* and has remained silenced in the domesticated rice accessions analyzed in this study.

Discussion: as all the results are for me not relevant or allowing any decisions, the whole discussion, especially the impact of domestication is for me to be changed.

We agree with the reviewer and have changed the wording of the title to more accurately reflect the purview and conclusions of the study. It now reads:

Tracking the origin of two genetic components associated with transposable element bursts during recent rice domestication.

Further, we have changed all references in the manuscript (including the discussion) to "key events" and refer instead to "genetic components". We agree with the reviewer that an "event" is something that actually happened (during domestication) as opposed to the interpretation that *Ping16A* and *Ping16A_Stow* may have been rare alleles in the wild progenitor that became more abundant in lineages leading to the bursting accessions.

Having said that, results of this study clearly demonstrate that *mPing* bursts are restricted to the very recent events previously reported in Lu et al 2017 - because no other *mPing* bursts were detected in the 3,000 accessions. Given the rarity of *mPing* bursts, it is reasonable to hypothesize that the bursts were initiated by all or a subset of the *Ping* loci present in these strains. Previously we showed that all bursting strains have (1) 7-10 *Ping16As*, and (2) *Ping16A_Stow*. In this study we advance the understanding of the association of *Ping16A_Stow* with high *mPing* copies (as described above). Further, this study advanced the understanding of the origin and function of *Ping16A* as follows: (i) that *Ping16A* is a variant of the original *Ping16G* which is the only *Ping* found in sequenced *O. rufipogon* accessions along

with all 4 *mPing* subtypes. Although *Ping16A* was not detected in the analyzed *O. rufipogon* accessions, it likely that it originated in *O. rufipogon*, and may be present as a rare allele.

REVIEWERS' COMMENTS:

Reviewer #1 (Remarks to the Author):

The authors have dealt with all of my concerns raised in my review of the manuscript. They have also gone a long way towards dealing with the concerns of Referee 3.

Reviewer #2 (Remarks to the Author):

The authors have accepted my criticism regarding the link made in the original version of the paper between Ping16A and Ping16A_Stow and the domestication process. However, their text still implies in places that events occurred 'during domestication' when their evidence only shows that these events occurred in the domesticated population, and cannot be associated with the domestication process itself. I therefore request that the following specific changes be made.

Title
Change to 'Tracking the origin of two genetic components associated with transposable element bursts in domesticated rice'.
The authors state in their rebuttal that 'mPing bursts are restricted to the very recent events', and by describing the bursts as occurring 'in domesticated rice' is consistent with this view as domesticated rice has, by definition, a recent origin.

Line 43
Change '…sustained activity of the mPing/Ping family during domestication…' to '…sustained activity of the mPing/Ping family in domesticated rice…'.

Line 174
Change 'first appeared during domestication ' to 'first appeared in domesticated rice'

Lines 279-280
Change 'during the hypothesized two domestication events that led to these subspecies' to 'during or after the hypothesized two domestication events that led to these subspecies'

Lines 283-284
Change 'significant proliferation during and after japonica domestication ' to 'significant proliferation during or after japonica domestication'

Terry Brown

Reviewer #3 (Remarks to the Author):

The current version of the manuscript has been widely improved, especially in terms of methods descriptions and clarification of ambiguous notions.
Thus, I think the current version is acceptable for publication.
I would like in addition to thank the authors for the huge work they provide in terms of clarifications and rewriting.

REVIEWERS' COMMENTS:

Reviewer #1 (Remarks to the Author):

The authors have dealt with all of my concerns raised in my review of the manuscript. They have also gone a long way towards dealing with the concerns of Referee 3.

Thank you for the supportive comments.

Reviewer #2 (Remarks to the Author):

The authors have accepted my criticism regarding the link made in the original version of the paper between Ping16A and Ping16A_Stow and the domestication process. However, their text still implies in places that events occurred 'during domestication' when their evidence only shows that these events occurred in the domesticated population, and cannot be associated with the domestication process itself. I therefore request that the following specific changes be made.

Title
Change to 'Tracking the origin of two genetic components associated with transposable element bursts in domesticated rice'.
The authors state in their rebuttal that 'mPing bursts are restricted to the very recent events', and by describing the bursts as occurring 'in domesticated rice' is consistent with this view as domesticated rice has, by definition, a recent origin.

The title has been changed accordingly.

Line 43
Change '…sustained activity of the mPing/Ping family during domestication…' to '…sustained activity of the mPing/Ping family in domesticated rice…'.

We have revised the sentence based on the suggestion. It now reads: "Finally, we show that sustained activity of the *mPing*/*Ping* family in domesticated rice produced the components necessary for *mPing* bursts, not the loss of epigenetic regulation." (Lines 32-34)

Line 174
Change 'first appeared during domestication ' to 'first appeared in domesticated rice'

We have revised the sentence based on the suggestion. It now reads: "the +16G/A *Ping* SNP and *Ping16A_Stow* could be detected in wild rice or first appeared in domesticated rice" (Lines 143-144)

Lines 279-280
Change 'during the hypothesized two domestication events that led to these subspecies' to 'during or after the hypothesized two domestication events that led to these subspecies'

We have revised the sentence based on the suggestion. It now reads: "The presence of the derived *Ping16A* in both *indica* and *japonica* accessions was initially confusing as it suggested the unlikely scenario that this variant arose independently during or after the hypothesized two domestication events that led to these subspecies." (Lines 253-256)

Lines 283-284
Change 'significant proliferation during and after japonica domestication ' to 'significant proliferation during or after japonica domestication'

We have revised the sentence based on the suggestion. It now reads: "Thus, *Ping16A* has experienced limited but significant proliferation during or after *japonica* domestication such that it now accounts for the majority of *Ping* elements present in domesticated rice accessions (Table 1)." (Lines 259-261)

Terry Brown

Reviewer #3 (Remarks to the Author):

The current version of the manuscript has been widely improved, especially in terms of methods descriptions and clarification of ambiguous notions.
Thus, I think the current version is acceptable for publication.
I would like in addition to thank the authors for the huge work they provide in terms of clarifications and rewriting.

Thank you for the supportive comments.
